# Spatial-temporal dynamics and influencing factors of archaeal communities in the sediments of Lancang River cascade reservoirs (LRCR), China

**DOI:** 10.1371/journal.pone.0253233

**Published:** 2021-06-15

**Authors:** Bo Yuan, Wei Wu, Mengjing Guo, Xiaode Zhou, Shuguang Xie

**Affiliations:** 1 State Key Laboratory of Eco-hydraulics in Northwest Arid Region, Xi’an University of Technology, Xi’an, Shaanxi, China; 2 State Key Joint Laboratory of Environmental Simulation and Pollution Control, College of Environmental Sciences and Engineering, Peking University, Beijing, China; Hohai University, CHINA

## Abstract

The spatial and temporal distribution of the archaeal community and its driving factors in the sediments of large-scale regulated rivers, especially in rivers with cascade hydropower development rivers, remain poorly understood. Quantitative PCR (qPCR) and Illumina MiSeq sequencing of the 16S rRNA archaeal gene were used to comprehensively investigate the spatiotemporal diversity and structure of archaeal community in the sediments of the Lancang River cascade reservoirs (LRCR). The archaeal abundance ranged from 5.11×10^4^ to 1.03×10^6^ 16S rRNA gene copies per gram dry sediment and presented no temporal variation. The richness, diversity, and community structure of the archaeal community illustrated a drastic spatial change. *Thaumarchaeota* and *Euryyarchaeota* were the dominant archaeal phyla in the sediments of the cascade rivers, and *Bathyarchaeota* was also an advantage in the sediments. PICRUSt metabolic inference analysis revealed a growing number of genes associated with xenobiotic metabolism and carbon and nitrogen metabolism in downstream reservoirs, indicating that anthropogenic pollution discharges might act as the dominant selective force to alter the archaeal communities. Nitrate and C/N ratio were found to play important roles in the formation of the archaeal community composition. In addition, the sediment archaeal community structure was also closely related to the age of the cascade reservoir and hydraulic retention time (HRT). This finding indicates that the engineering factors of the reservoir might be the greatest contributor to the archaeal community structure in the LRCR.

## 1 Introduction

The river reservoir system is an important link for the transportation of various biogenic elements to the ocean in the basin surface ecosystem [1–3]. Large-scale dams and reservoirs are built on rivers to meet the needs of increasing social and economic development [4–7]. The interception of the dam changes the hydrodynamic process of the river flow, blocking and depositing the sediments and increasing the risk of eutrophication of the reservoir water, which may lead to a change in the biogeochemical cycle of the source material and the corresponding environmental problems [8–11]. Compared with the overlying water of the reservoir, the sediment of the reservoir is considerably rich in environmental information regarding regional geological structure-activity, geological environment background, climate change, and human activities. At the same time, the physical, chemical, and biological characteristics of the reservoir sediment remain relatively stable for a short period, which can allow for the "recording" of the disturbance of the reservoir [12–14]. Researchers aim to reveal the characteristics of the reservoir environment, biological succession, and the migration and transformation of pollutants.

There are many microbial communities in river sediments, and about 6–40% of prokaryotes on earth exist in the sediment environment [15]. The sediment archaeal community, as an important part of sediment microbiology, can not only exist in extreme conditions such as under high temperature, acidity, alkalinity, salinity, and anoxic conditions but can also survive in the common water ecological environment [16–18]. At the same time, sediment archaeal communities are important participants in biogeochemical processes such as the carbon cycle, nitrogen cycle, and sulfur cycles. However, most archaeal communities are difficult to cultivate, which limits the understanding of the physiological and biochemical mechanisms, genetic characteristics, and ecological functions of the archaeal community. With the vigorous and innovative development of new molecular biology techniques, researchers have made a breakthrough in understanding the archaeal community. The archaeal community has increased from the first two phyla to the current 28 phyla. The newly discovered archaeal communities are widely distributed in marine, estuarine, river, wetland, pond, lake, and reservoir ecosystems [18–22]. At present, the study of Archaea in different environments has become one of the hotspots of biogeochemistry.

The Lancang-Mekong River (the upper reach in China is generally called the Lancang River) [23] is the largest and most controversial international river in the Asian continent and the Mother River of Southeast Asian countries [24–26]. The Yunnan section of the Lancang River is abundant and sufficient in hydropower resources and is the key river for cascade hydropower resource development in the southwest of China [27]. In the early 1990s, the Manwan hydropower station, the first hydropower station in the mainstream of the Lancang River, was built. By the beginning of 2018, seven cascade hydropower stations were built and operated in the Lancang River, and the Yunnan section of the Lancang River has become a typical water storage river [28]. Although the construction of cascade reservoirs has brought considerable hydropower benefits to the home country, from the perspective of the river basin, they have also deprived tens of thousands of people living on the water downstream. At the same time, the impact of cascade dams on regional biodiversity reduction and ecosystem service value is inevitable [29]. The pressure of cascade dam construction on river ecological security may become the trigger point of cross-border resource and environmental conflict and regional geopolitical crisis [30, 31]. To accurately evaluate the ecological environmental impact of cascade dams, it is necessary to comprehensively explore the disturbance mechanism of biological and non-biological factors from multiple perspectives, and this is undoubtedly a very effective way to study the characteristics and ecological functions of microbial communities in sediments.

Previous studies have shown that the macrozoobenthic communities have been altered, and the dominant species have transformed from lotic to lentic in the regulated Lancang River [27]. At the same time, the fish species decreased significantly and the vegetation landscape pattern fragmentation increased after the construction of the Lancang River Cascade dam, which further confirmed the adverse impact of dam construction [32, 33]. However, most of the existing studies focused on the characteristics of bacterial community variation and biogeographical driving factors [28], while few have focused on the changes in archaeal communities in Lancang River sediments. Sediment archaeal is the basic participant in the biogeochemical cycle of biogenic materials and is also the main driving force of reservoir water quality changes. At present, large-scale dam construction in the upper reaches of the Lancang River is still ongoing at an unprecedented speed, which means that the scale and scope of potential environmental and ecological impacts are extremely complex and uncertain [31], and it is urgent to fully understand the impact of cascade dams on river ecosystems and biogeochemical cycles. Based on this, quantitative PCR (qPCR) and Illumina MiSeq high-throughput sequencing systems were adopted to analyze the spatial-temporal characteristics of the abundance, richness, diversity, and composition of archaeal communities in the sediments of the cascade reservoirs, and to explore the environmental factors driving these changes. This study is of great significance to understand the microbial ecology in the Lancang River Cascade Reservoirs (LRCR) and reveal the structural characteristics of archaeal communities in the sediments of the Lancang River in the context of cascade development. At the same time, it can provide foundational information and a theoretical basis for ecological security management of reservoirs.

## 2 Material and methods

### 2.1 Site description

**Fig 1:**
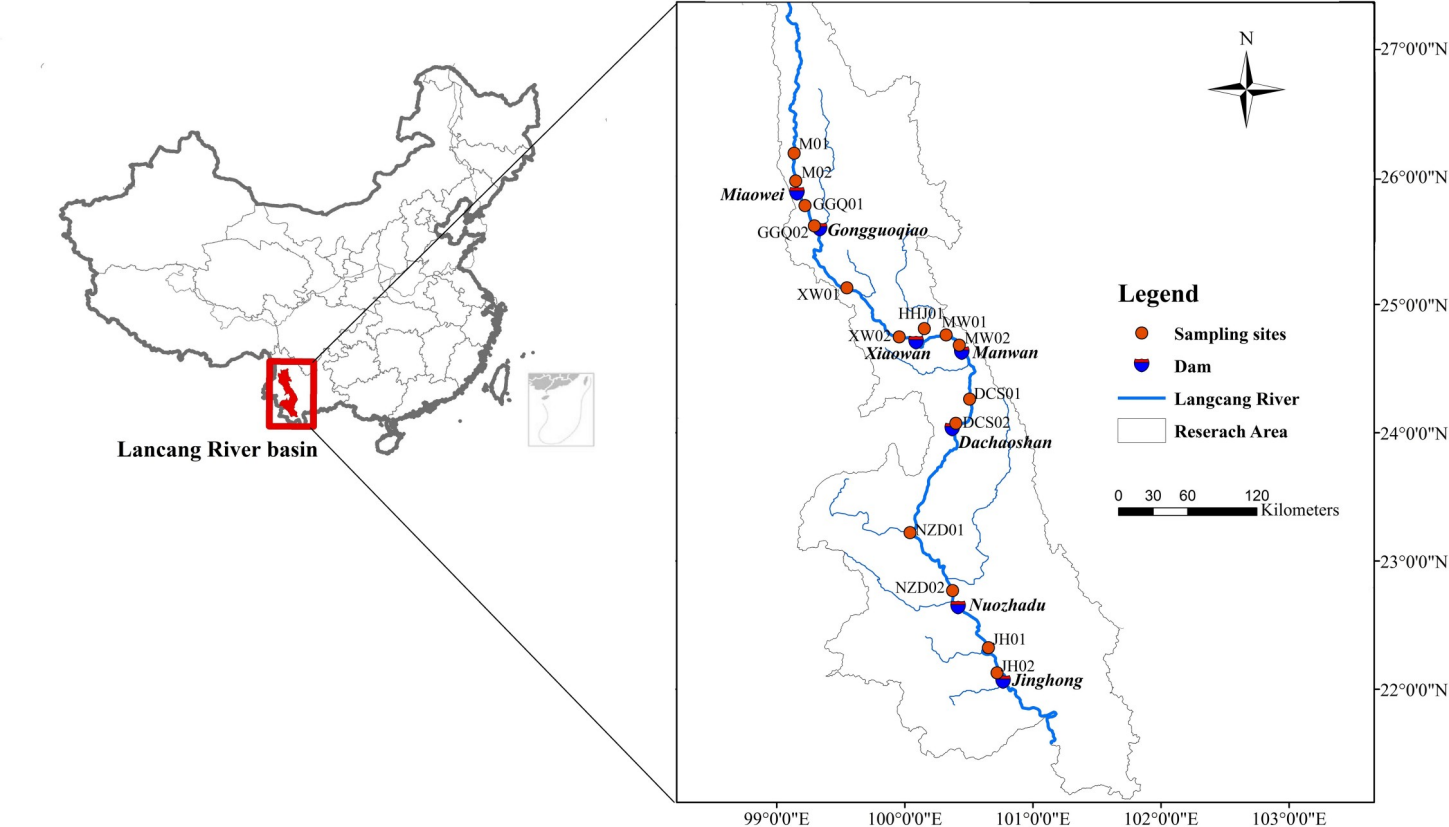
Study area and sampling sites in LRCR (the base map for this figure originated from NASA earth observatory (public domain)).

The Yunnan section of the Lancang River is characterized by a monsoon climate, which is characterized by wet (November to April) and dry (May to October) seasons. Approximately 85% of the annual precipitation is concentrated during the wet season. By 2018, there were seven cascade hydropower stations in the Yunnan section that were built and stored water for power generation (Fig 1). The hydropower stations along the Lancang River flow direction are Miaowei, Gongguoqiao (GGQ), Xiaowan (XW), Manwan (MW), Dachaoshan, Nuozhadu (NZD), and Jinghong (JH) hydropower stations, respectively (Fig 1). The basic engineering features of the cascade hydropower stations are shown in S1 Table.

### 2.2 Sample collection and pretreatment

In this study, sediment samples were collected in the summer (August 12–24, 2018) and winter (January 15–27, 2019) of the Lancang River longitudinally along the seven hydropower stations. Our on-site investigation and two sample collection campaigns in the study area were approved by Huaneng Lancang River Hydropower Co., Ltd. The longitude, latitude, and altitude information of each sample site was determined by a GPS (S2 Table), and the surface sediment samples (0–3 cm) at each site (three parallel samples) were obtained from 3–5 areas (within 80 cm × 80 cm) by a Peterson dredger and placed in aseptic polyethylene bags. After the sampling time and water depth of each site were marked, the samples were quickly placed in a vehicle refrigerator and stored in dark conditions. A proper amount of sample was taken and sub-packed in a 50 ml centrifuge tube and stored in liquid nitrogen for subsequent molecular biological analysis; the remaining sediment samples were refrigerated at 4°C and brought back to the laboratory as soon as possible for deposition physical and chemical properties analysis. The sediments brought back to the laboratory were dried using a vacuum freeze-drying machine, ground, screened (100 mesh), placed in a dense bag, sealed, and stored at 4°C until use.

### 2.3 Physicochemical properties analysis

To determine the pH and electronic conductivity (EC), the sediment was first mixed with ultrapure water without CO_2_ at a volume ratio of 1:5 before the test, and then the supernatant was collected after standing for a while and measured by thermoelectric corporation (Beverly, MA, USA). The moisture content of the sediment was determined using the weight method (drying method). Specifically, the sediment with known weight was placed in an oven at 105°C and dried to a constant weight. The ratio of the mass difference before and after drying to the mass before drying was used to calculate the moisture content. After the effective leaching of carbonate in sediment samples with 3 M HCl, the content of total carbon (TC) and total organic carbon (TOC) [31] was determined using an elemental Vario TOC analyzer (Frankfurt, Germany). The grain size of the sediments was measured using a laser particle size analyzer (Mastersizer 2000; Malvern Co., UK). The total nitrogen (TN) content in the sediment was measured using the Kjeldahl digestion method, and the total phosphorus (TP) content was measured using the molybdenum blue colorimetric method. Sediment ammonia nitrogen (NH_4_^+^-N), nitrate-nitrogen (NO_3_^—^N), and nitrite-nitrogen (NO_2_^—^N) contents were extracted from fresh sediment using 2 M KCl for 1 h, filtered through a 0.45 μm polyethersulfone aqueous phase filter membrane, and measured using a continuous flow analyzer (SKALAR SAN ++, the Netherlands). Three parallel samples were set to determine the physical and chemical factors of all the samples.

### 2.4 DNA extraction and high-throughput sequencing

Total genomic DNA was extracted from 0.5 g fresh sediment samples using the PowerSoil™ DNA Isolation Kit (MOBIO, USA), according to the manufacturer’s protocols. The quality and quantity of extracted DNA were determined using a Picodrop microliter spectrophotometer (Picodrop, Saffron Walden, Essex, UK). The total genomic DNA fragment (425 bp) of each sample was amplified using the specific archaeal primer Arch524F (5-TGYCAGCCGCCGCGGTAA-3) / Arch958R (5-YCCGGCGTTGAVTCCAATT-3) [34]. The PCR conditions using Arch524F / Arch958R were as follows: initial denaturation at 95°C for 3 min followed by 35 cycles at 95°C for 45 s, annealing at 55°C for 45 s, and 72°C for 1.5 min; and 72°C for 10 min. The products were stored at 4°C. The PCR products were detected by electrophoresis on a 2% agarose gel. The AXYGEN gel recovery kit was used to recover the target fragment. The PCR products were detected and quantified using a microplate reader fluorescence quantitative system (Bio Tek, FLx800, USA) using the fluorescent reagent in the Quant-iT PicoGreen dsDNA Assay Kit. Then, according to the results of fluorescence quantification and the sequencing demand of each sample, each sample was mixed in proportion. The sequencing library was prepared using the TruSeq Nano DNA LT Library Prep Kit (Illumina). After the library was confirmed to be qualified, Shanghai Personal Biotechnology Co., Ltd. (Shanghai, China) was commissioned to carry out double-terminal sequencing analysis using an Illumina MiSeq PE250 sequencing platform.

### 2.5 Quantitative PCR (qPCR)

The abundance of the archaeal 16S rRNA gene was quantified by qPCR using the same primers used for high-throughput sequencing. The qPCR was performed using a Bio-Rad CFX96 System (Bio-Rad Laboratories Inc., Hercules, CA, USA) and AceQ qPCR SYBR Green Master Mix quantitative PCR reagents. The 15-μl reaction mixture system included 2× SYBR Green mix (7.5 μl), 0.7 μl Arch109F and Arch915R primers, template DNA (1 μl), and ddH_2_O (15 μl). The amplification reaction conditions were as follows: 5 min at 95°C followed by 40 cycles of 95°C for 10 s, annealing for 60 s at 60°C, and extension at 72°C for 30 s. Melting curve analysis was carried out from 60 to 95°C. Standard curves ranging from 10^3^ to 10^9^ gene copies/mL were obtained using serial dilutions of linearized plasmids (pGEM-T, Promega) containing cloned 16S rRNA genes amplified from environmental DNA. The average amplification efficiency and coefficient (R^2^) of the archaeal 16S rRNA gene were 87.48% and 0.9977, respectively.

### 2.6 Bioinformatics and statistical analysis

The raw Illumina reads were merged using FLASH (v1.2.7, http://ccb.jhu.edu/software/FLASH/) and further processed according to the protocol [35]. QIIME (version 1.8.0, http://drive5.com/uparse/) was used to merge and divide the OTUs according to 97% similarity. OTUs with an abundance of less than 1% of the total sequencing amount of the whole sample was removed. The sequence with the highest abundance in each OTU was selected as the representative sequence of the OTUs. Then, the representative sequences of each OTU were compared with the Greengenes database, and the annotation proportion and species relative abundance of each OTU classification level was obtained [36]. To avoid the deviation caused by different sequencing depths, the data of each sample were homogenized according to the sample with the least amount of data. Using the MOTHUR software, the statistical algorithm of Metastats (http://metastats.cbcb.umd.edu/) [37] was used to test the sequence size (i.e., absolute abundance) difference between the door and genus-level classification units in the sample (Group). The Mothur software was used to call the statistical algorithm of Metastats (http://metastats.cbcb.umd.edu/) to test the sequence size (i.e., absolute abundance) difference between each taxon at the gate and genus levels.

One-way analysis of variance (ANOVA) followed by the Student-Newman-Keuls test was used to check for significant differences (P < 0.05) in the physicochemical properties and the abundance of archaeal 16S rRNA genes among all sampling sites. SPSS 22.0. (IBM, Armonk, NY, USA) with Spearman rank, correlation analysis was used to examine the potential links between the determined physicochemical variables and the abundance, richness, and diversity of archaeal communities. Nonmetric multidimensional scaling (NMDS) analysis based on the Bray-Curtis distance was calculated to describe the structural distribution of the archaea community and the similarity between different samples. Metastats were performed to compare the archaeal communities at different sampling sites to detect different abundant taxa at the phylum and genus levels. Phylogenetic Investigation of Communities by Reconstruction of Unobserved States (PICRUSt) was used to predict the metabolic function of archaeal communities [38]. Detrended correspondence analysis (DCA) was used to determine a suitable ordination analysis method. The longest DCA axis was found to have a gradient length of less than three standard deviation units, so redundancy analysis was performed with Monte Carlo tests using CANOCO 5.0 (Biometrics Wageningen, The Netherlands) and R software (version 3.4.2, vegan package).

The gene sequences obtained from Illumina MiSeq sequencing in this study were submitted to the NCBI short-read archive under the accession number PRJNA595475.

## 3 Results

### 3.1 Physicochemical properties of sediments in cascade reservoirs

S3 Table shows the physicochemical parameters of sediments in LRCR. The pH values of sediments ranged from 6.99 to 8.44, and the average pH values of sediments in summer and winter were similar; the spatial differences were not significant (P > 0.05). The water content of sediments ranged from 16.9% to 28.3%, with no significant differences spatially (P > 0.05) but significant seasonal differences (P < 0.01). The physical characteristics of surface sediments in most sampling sites were mainly composed of fine sand, clay, and silt, and the highest percentage of silt in the sediments was 50.9%. The TOC content in sediments ranged from 9.51 to 20.96 g/kg, with higher values in the reservoir and lacustrine zones than in the transitional zones for most of the sampling sites. The TP content in sediments ranged from 439.0 to 961.1 mg/kg; TP content was significantly different spatially (*P* < 0.01); however, the difference between summer and winter was not significant (*P* > 0.05). The TN content in sediments ranged from 647.8 to 1888.5 mg/kg; the TN content was higher in the lake section than in the transition section, exhibiting a very high significant difference spatially (*P* < 0.01). The NH_4_^+^-N content in sediments ranged from 12.7 to 33.0 mg/kg, with higher values in summer than in winter. The NO_3_^—^N content ranged from 1.9 to 4.5 mg/kg and was significantly positively correlated with the NH_4_^+^-N concentration (R = 0.545, *P* < 0.01, n = 30).

### 3.2 Abundance of the archaeal community

**Fig 2:**
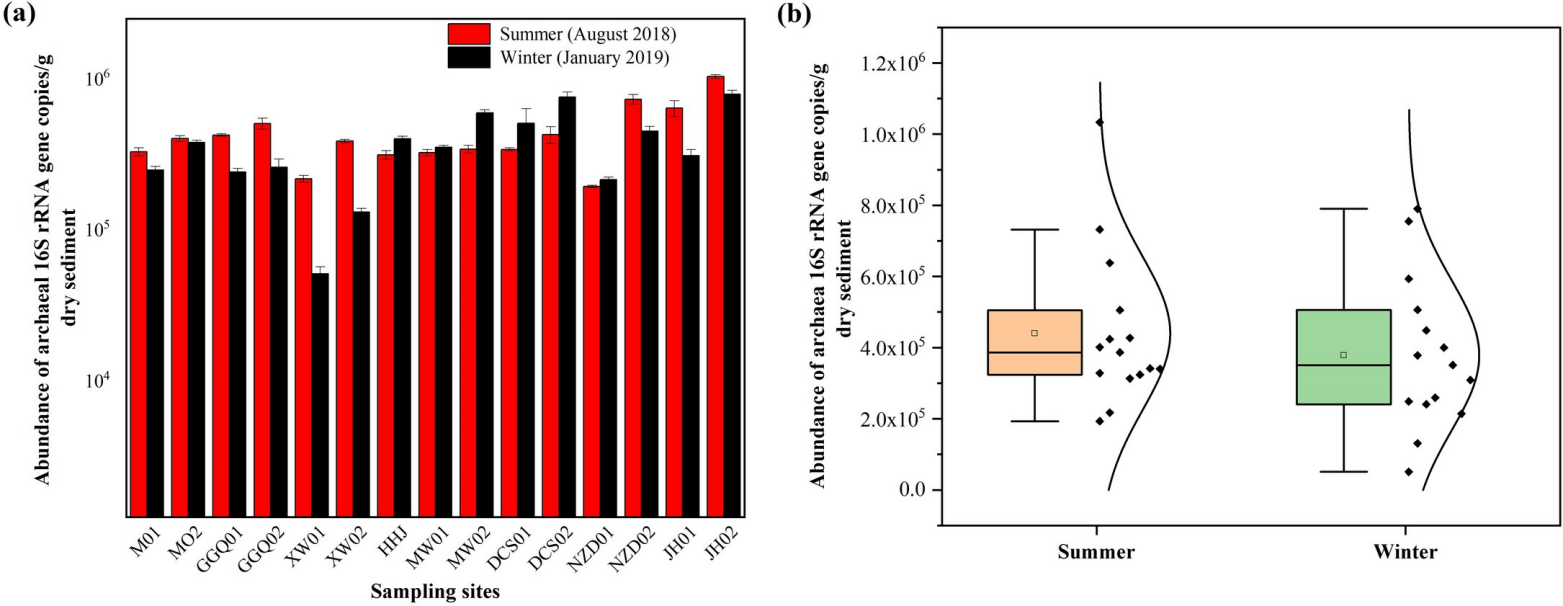
The abundance of archaeal 16S rRNA gene in summer and winter sediments of LRCR.

A large variation in the abundance of archaeal species was observed in sediments of the LRCR (Fig 2). The abundance of the archaeal 16S rRNA gene in sediments of cascade reservoirs ranged between 1.93 × 10^5^ and 1.03 × 10^6^ copies per gram dry sediment, with a mean value of 4.40 ± 0.26 × 10^5^ copies per gram dry sediment in summer (wet season), while the archaeal abundance of the sediment samples in winter (dry season) ranged from 5.11 × 10^4^ to 7.90 × 10^5^ copies per gram dry sediment, with a mean value of 3.78 ± 0.31 × 10^5^ copies per gram dry sediment. The abundance of the archaeal community was slightly higher in summer than in winter, in addition to SHHJ01, SMW01, SMW02, SDCS01, and SDCS02. In the longitudinal cascade reservoirs, the abundance of archaeal communities fluctuated and increased in the upper reaches of the DCS reservoir in summer, while the abundance of the archaeal community increased significantly in the NZD and JH reservoirs. As for the cascade reservoirs, the 16S rRNA gene abundance per gram of sediment (dry weight) in summer was not significantly different from that in the winter (P > 0.05). The spatial variation of archaeal abundance in the cascade reservoir sediment fluctuated slightly (P < 0.01).

### 3.3 Richness and diversity of the archaeal community

**Fig 3:**
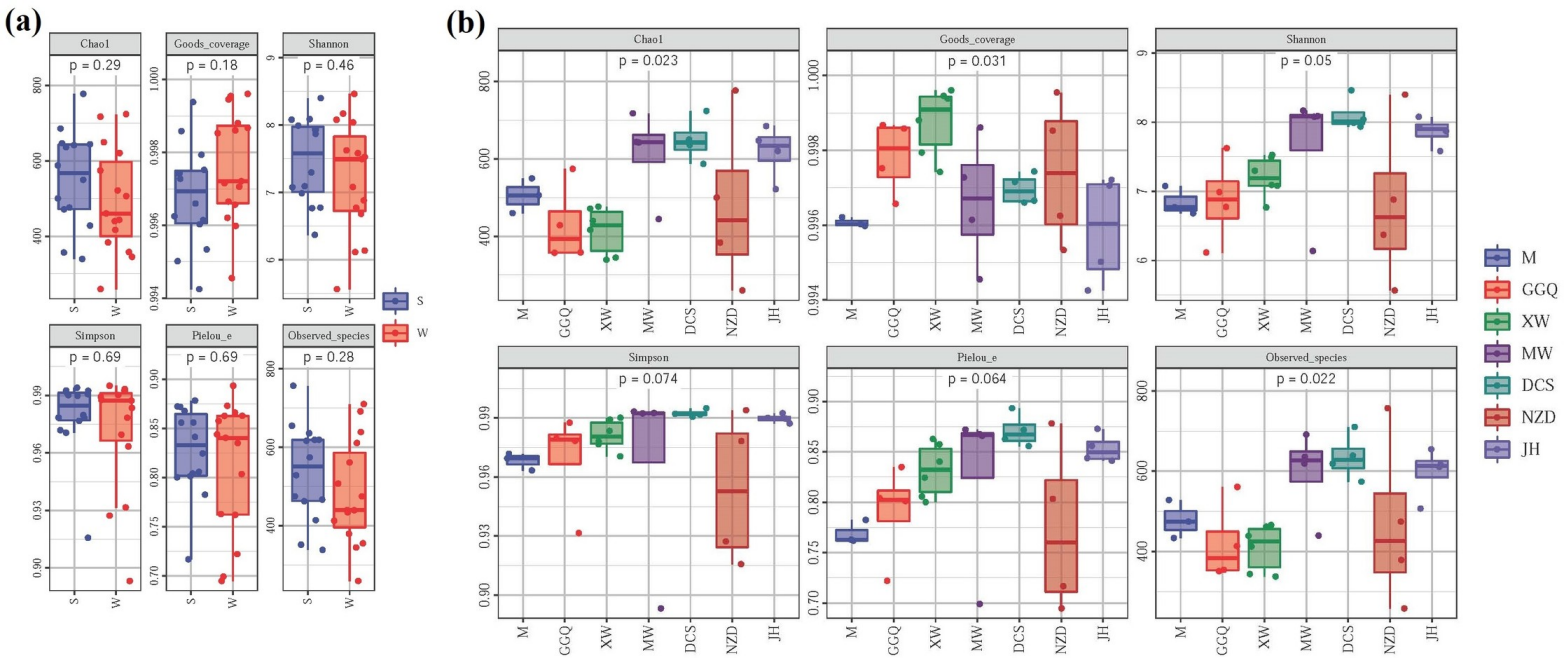
Archaeal community richness and diversity of LRCR in different seasons (a) and reservoirs (b).

In this study, the obtained high-quality archaeal sequences from each sample ranged between 29,085 and 37,625, normalized to 33,558, to compare archaeal community richness and alpha diversity. High Good’s coverage (≥97%) illustrated that the OTUs of each sediment archaeal library were well captured. All the normalized sediment archaeal sequences in summer and winter could be grouped into 8,226 OTUs (S1 Fig). The number of archaeal OTUs in the sediment of the LRCR was 3380–7791 in summer and 2044–7299 in winter. No obvious temporal variation in OTU of archaeal richness was observed in summer and winter. However, at some sampling sites, the summer sediment samples had more OTUs than the corresponding winter samples. A strong overlap of OTU of archaeal richness was observed in different reservoirs. The Chao1 indices of the sediment archaeal community in the summer and winter were 960–2414 and 543–2300, respectively (Fig 3). In most sampling sites, archaeal community richness was higher in summer than in winter. Moreover, sediments in downstream reservoirs had much higher OTU numbers and Chao1 indices than those of the upstream reservoirs. However, in either summer or winter, the evident spatial variation of sediment archaeal OTU number and Chao1 richness did not emerge in these seven reservoirs. The Shannon diversity indices of sediment archaeal communities in summer and winter were 6.97–9.25 and 5.37–8.68, respectively. The Simpson diversity indices of sediment archaeal communities in summer and winter were 0.970–0.993 and 0.882–0.986, respectively. The results of the one-way ANOVA showed that the indices of richness and diversity in summer and winter were almost identical, so seasonal effects on the archaeal community richness and diversity were not significant. However, archaeal diversity in the LRCR demonstrated obvious spatial variation.

### 3.4 Taxonomic structures and composition of the archaeal community

**Fig 4:**
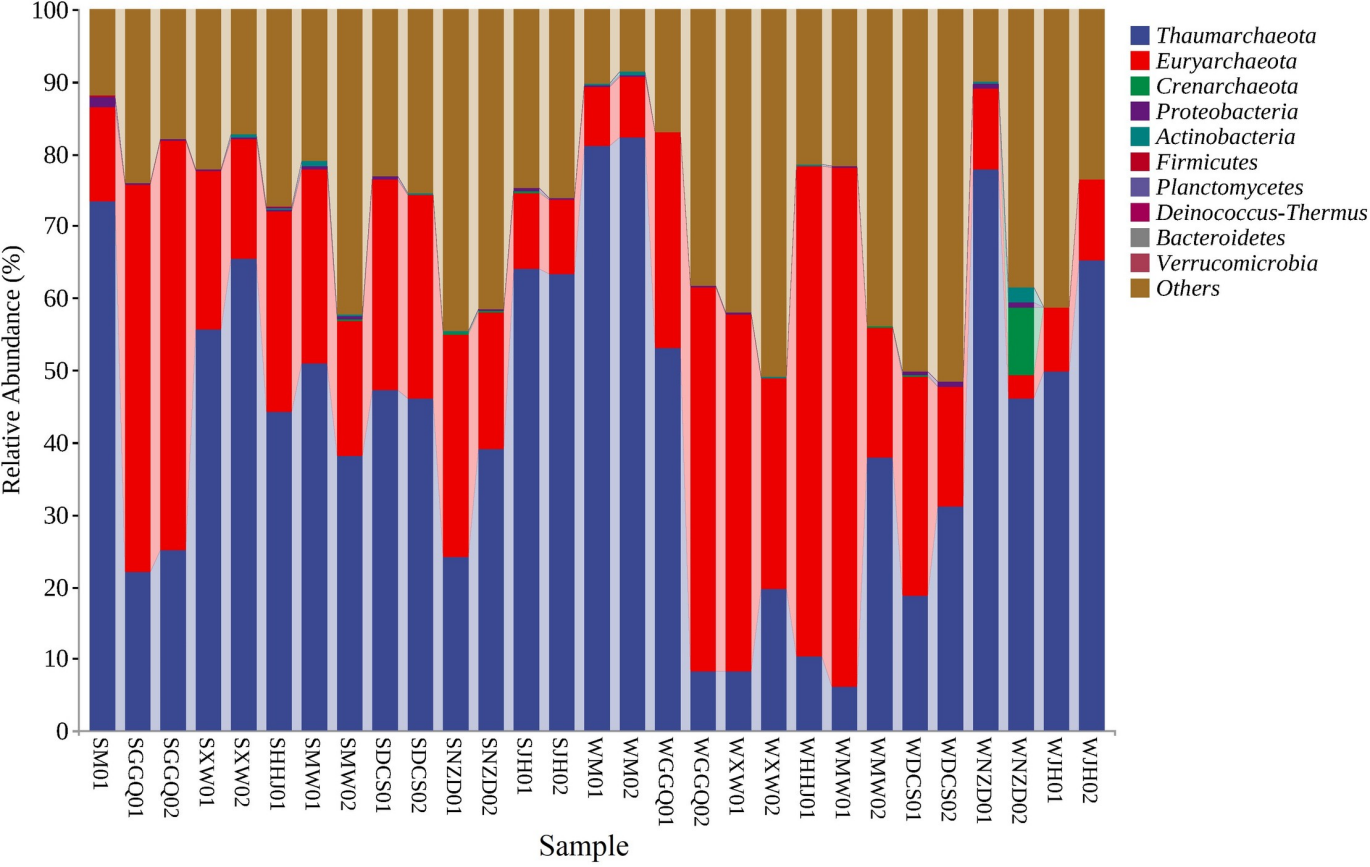
Archaeal phyla relative distribution in LRCR.

In the present study, *Thaumarchaeota* was the predominant archaeal phylum in the summer (4.94–86.13%) and winter (18.97–79.89%) of the sediment samples from the LRCR (Fig 4). In both seasons, the relative proportion of sediment *Thaumarchaeota* organisms showed a remarkable spatial variation in these seven cascade reservoirs. Moreover, at a given sampling season in the LRCR, the winter sediment sample generally showed a lower *Thaumarchaeota* proportion than the corresponding summer sediment sample. Overall, the relative proportion of *Thaumarchaeota* in the sediments of the upstream cascade reservoirs was lower than that of the downstream; in winter, the relative proportion of *Thaumarchaeota* in the sediments of the GGQ, XW (including HHJ01), MW, and DCS reservoirs was significantly lower than that in summer, while *Thaumarchaeota* had an absolute advantage and did not show significant changes in summer and winter in the M reservoir, which was newly built upstream of the Lancang River. *Euryarchaeota* was the second largest phylum in the sediments of cascade reservoirs, which comprised a considerable proportion of 11.19–63.03% in the summer and 3.63–77.91% in the winter. *Crenarchaeota*, as the third-largest archaeal phylum, comprised a relatively high proportion in the summer (3.5%) and winter (1.10–46.81%). The relative proportions of *Euryarchaeota* and *Crenarchaeota* in the upper and middle reaches of the cascade reservoir samples were lower in the summer than in the winter, while that in the lower reaches of the reservoir was the opposite, which indicated that the seasonal variation in the relative proportion distribution of *Euryarchaeota* and *Crenarchaeota* in the sediment is different among the cascade reservoirs. In addition, small proportions of other archaea organisms, such as *Asgardaeota* (0.76%) and *Nanoarchaeota* (0.01–0.22%), were also found in Lancang River sediments.

**Fig 5:**
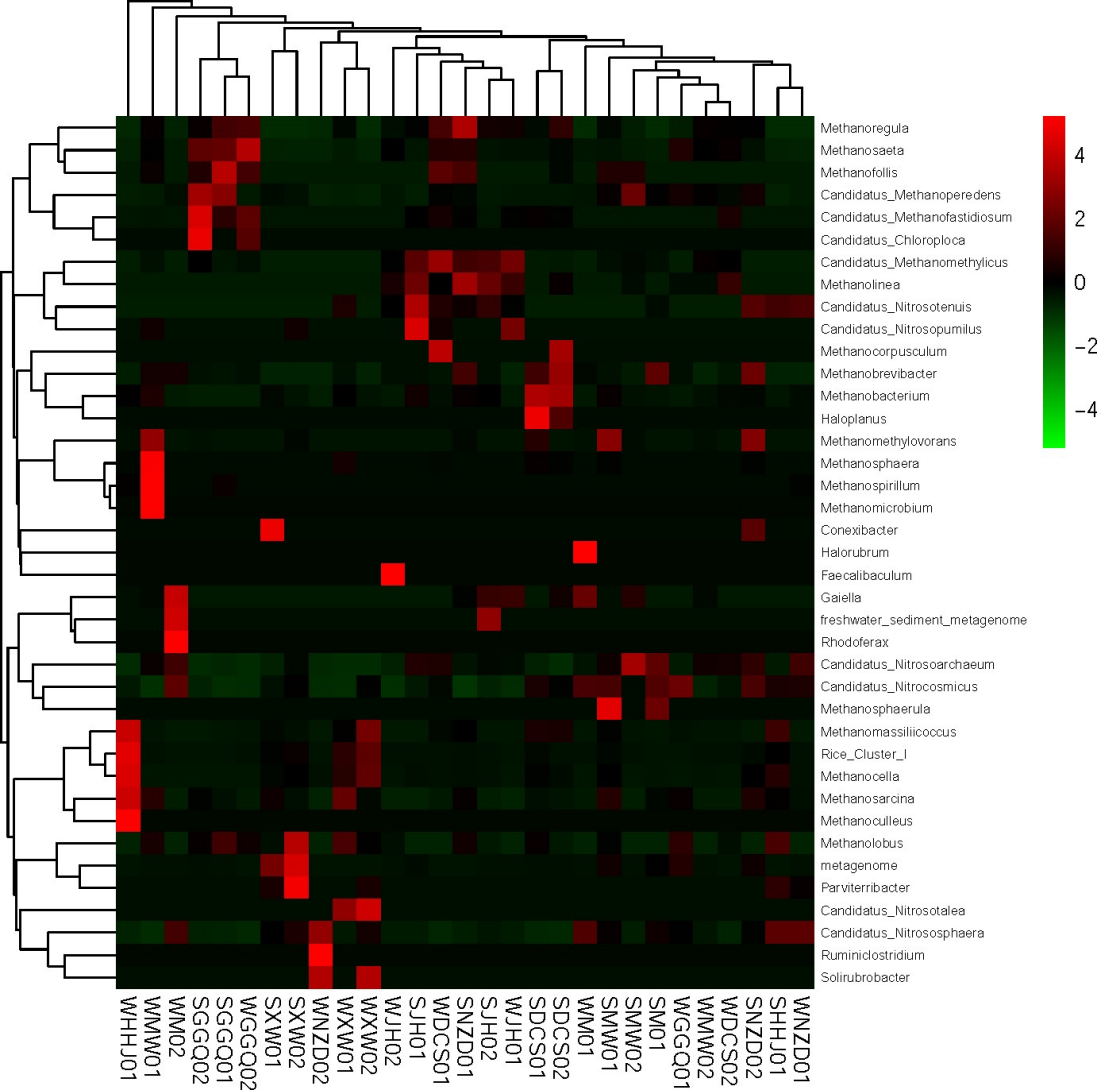
Relative abundance of the major archaeal genera from LRCR.

To explore the main microorganisms and their functions in the sediment archaeal community of the LRCR, the first 50 genera of abundance were screened (Fig 5). Based on the cluster analysis of the selected dominant genus, there was no obvious cluster of Archaea in different seasons and different reservoirs, which showed that the community structure of Archaea is quite different in different habitats. Because most archaea are not cultured, there are only four classes of Archaea that can be classified into genera in this study: *Methanomicrobia*, *Nitrososphaera*, *Methanobacteria*, and *Verstraetearchaeota*. *Methanosaeta*, *Methanosarcina*, and DAMO Archaea (*Candidatus Methanoperedens*) accounted for the most, among which *Methanosaeta* accounted for 0.01–50.92%, with an average of 8.35%. *Methanosarcina* accounted for 0–40.25%, with an average of 5.80%. *Candidatus Methanoperedens* accounted for 0–22.5%, with an average of 4.19%. *Rice cluster I* only accounted for a large proportion of the XW reservoir sampling sites in winter. Three types of ammonia-oxidizing archaea related to the nitrogen cycle were found under *Nitrososphaera*, namely *Candidatus Nitrososphaera*, *Candidatus Nitrosocosmicus*, and *Candidatus Nitrosoarchaeum*, which accounted for 0.2–0.84%, 0–1.10%, and 0–0.70% of the sediment, respectively.

**Fig 6:**
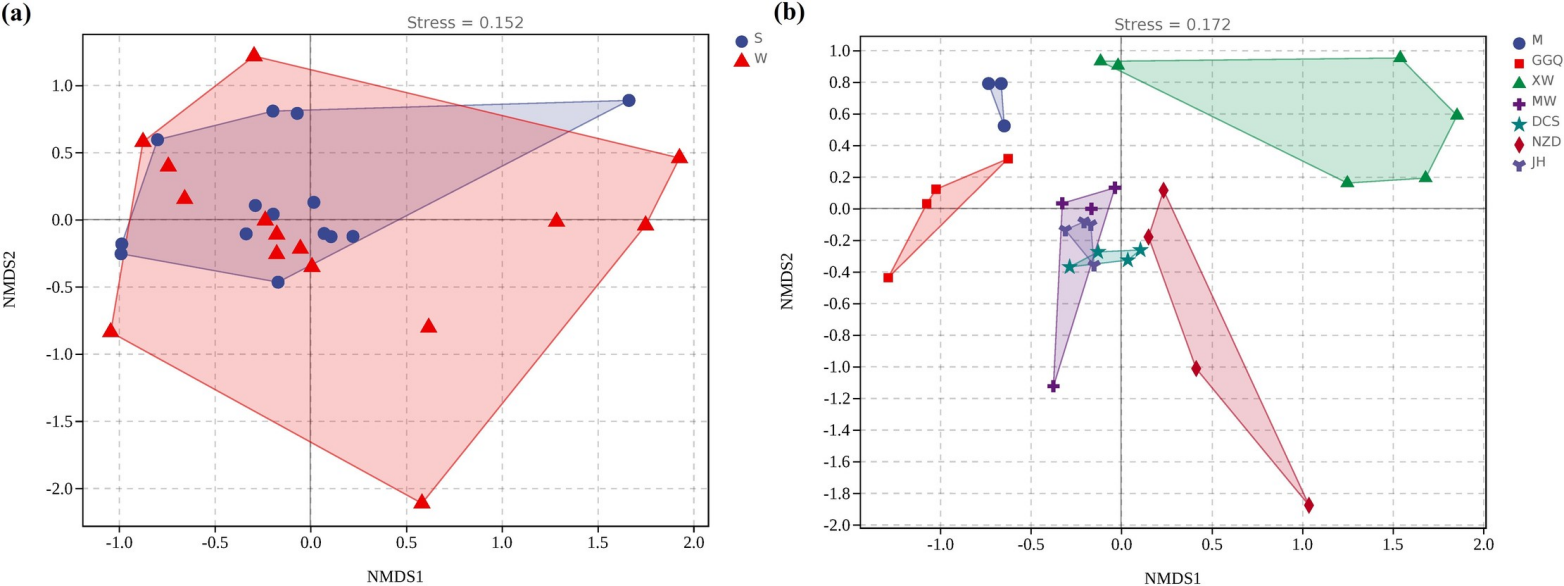
Archaeal community compositions of sediment samples as indicated by NMDS in different seasons (a) and reservoirs (b).

The hierarchical clustering analysis based on the 16S rRNA archaeal gene indicated that seasonal variation had no significant effect on the structure of archaea communities, while the location of cascade reservoirs and reservoir engineering characteristics showed remarkable spatial heterogeneities (S1 Fig). Similarly, the results of the NMDS analysis showed that the 16S rRNA archaea gene was clustered in different quarters (Fig 6). Each point represents a sample, and points of different colors belong to different samples (groups). The closer the distance between the two points, the higher the similarity of the microbial community structure between the two samples and the smaller the difference. Some of the XW and NZD sampling sites were far away from other sampling sites, which were clustered separately, indicating that the sediment archaeal community in the deep-water reservoir was remarkably different from that in the shallow-water one.

### 3.5 Metastats analysis of archaeal communities in sediment

Pairwise comparisons were performed using the Metastats analysis of sediment samples to determine the spatial and temporal variability of archaeal communities. It was found that 1 (grouping of winter and summer) and 5 (grouping of cascade reservoirs) phyla in different sediment sample groups showed significant differences (S4 and S5 Tables). The community structure of the two groups was very different at the phylum level (); however, significant differences were found in 30 genera (*P* < 0.05). The Metastats test identified several archaeal taxa with significant differences, such as *Asgardaeota*, *Crenarchaeota*, *Diapherotrites*, *Euryarchaeota*, *Nanoarchaeaeota*, and *WS1* at the phylum level (S2 and S3 Figs). High variation was also observed in *Candidatus Chloroploca*, *Candidatus Methanofastidiosum*, *Candidatus Methanomethylicus*, and *Candidatus Methanoperedens* genera (S4 and S5 Figs).

### 3.6 Prediction of archaeal metabolism function

PICRUSt (phylogenetic investigation of communities by reconstruction of unobserved states) is a novel tool for predicting the metabolic function of archaeal communities, which can predict the 16S rRNA gene sequence in the KEGG functional database and divide metabolic pathways into six categories. The annotations were classified into 274 functional KEGG results at level 3, including various metabolites, biochemical reactions, enzymes, and genes. The metabolic genes and pathways related to the degradation of xenobiotic pollutants, carbohydrates, nitrogen, phosphorus, and metabolism were investigated in this study.

Among the xenobiotic pollutants in our study, the gene abundance-related nitrotoluene degradation was the highest, showing fluctuations and decreases in the sediments of cascade reservoirs along the river direction, and the trend was the same in summer and winter, indicating the interception effect of dams. The number of genes affiliated with benzoate degradation, chloroalkane, and chloroalkene degradation, and toluene degradation were also highly abundant, and the variation trend along the longitudinal direction of the cascade reservoirs was consistent. Polycyclic aromatic hydrocarbon (PAH) degradation-related genes are different in different reservoirs and seasons. The abundance of PAH-related genes was highest in the HHJ in winter and decreased gradually in the downstream reservoirs. Benzoates and aminobenzoates are the key intermediates of PAH biodegradation, and genes related to benzoate and aminobenzoate biodegradation also had a large proportion in all samples in the two seasons (S6 Table). Naphthalene is also a PAH and naphthalene degradation gene, which has a greater advantage in M and NZD reservoirs. The results showed that seasonal variation had little effect on the major biogenic elements of C, N, P, and S, and the gene abundance related to nitrogen metabolism was the highest; the gene abundance related to carbohydrate metabolism and metabolism was similar. The abundance of genes related to phosphorus metabolism was the lowest and varied greatly in different reservoirs. The abundance was higher in the M reservoir and XW and NZD reservoirs, which indicated that the location and regulation performance of the dam had a great influence on sediment interception.

### 3.7 Influential factors regulating the archaeal community

**Fig 7:**
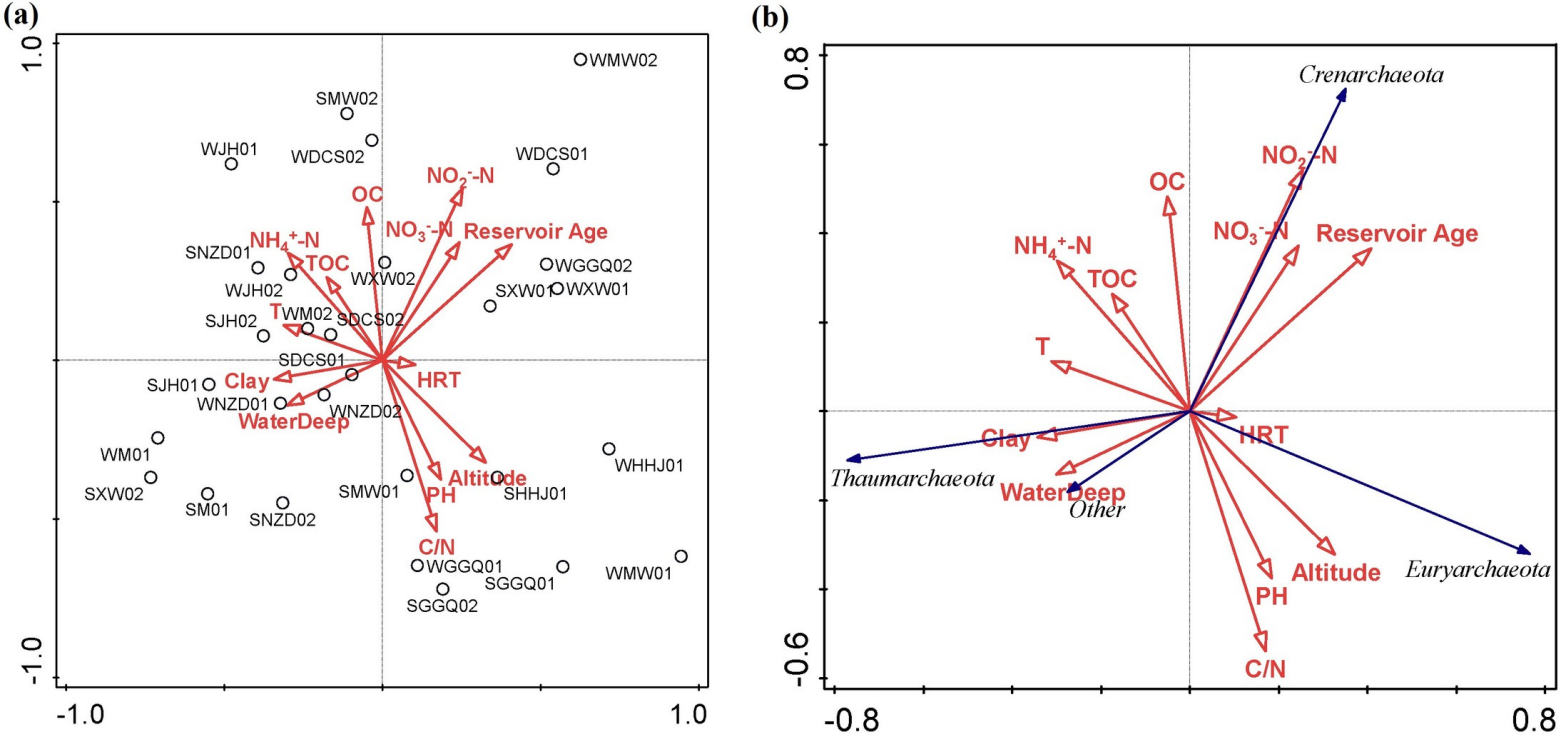
RDA ordination plot for the first two principal dimensions of archaeal communities and environmental factors.

SPSS 22.0 was used to analyze the correlation between the archaeal community and physical-chemical properties in the sediments of the cascade reservoirs, as shown in Table 1. Spearman correlation analysis showed that archaeal community abundance was positively correlated with water temperature, sediment silt content, organic matter, TP, and TN (*P* < 0.05), but negatively correlated with sediment sand content and altitude (*P* < 0.05). There was a significant positive correlation (*P* < 0.05) between the OTU number, Shannon diversity, Simpson index, and Chao 1 richness and water temperature, but negative correlations with C/N and altitude (*P* < 0.05). For the analysis of archaeal phylum, *Thaumarchaeota* proportion was negatively correlated with reservoir age (*P* < 0.05); *Euryarchaeota* proportion was negatively correlated with NH_4_^+^-N content (*P* < 0.05), while *Crenarchaeota* proportion was positively correlated with NO_2_^—^N and reservoir age (*P* < 0.05). For the analysis of archaeal classes, the *Nitrososphaera* proportion was negatively correlated with reservoir age (P < 0.05), *Methanobacteria* was positively correlated with the pH of the sediments and altitude (*P* < 0.05). The *Bathyarchaeota* proportion was positively correlated with the NO_2_^—^N and reservoir age (*P* < 0.05); *Methanobacteria* was positively correlated with the silt content and pH of the sediments, while the proportion of Thermoplasmata was positively correlated with HRT. For the analysis of the archaeal genus, *Methanosaeta* was negatively correlated with water depth and HRT (*P* < 0.05 or *P* < 0.01); *Methanosarcina* was negatively correlated with temperature, silt content, OC, and TN content of the sediment (*P* < 0.05, *P* < 0.01), and positively correlated with reservoir age; *Candidatus Methanoperedes* showed a positive correlation with water depth and a negative correlation with reservoir age (*P* < 0.05); *Methanobacterium* showed a positive correlation with sand content and reservoir age (*P* < 0.05) and a negative correlation with silt content, pH, OC, and altitude (*P* < 0.05). In addition, *Candidatus Nitrososphaera* related to the ammonia oxidation process was positively correlated with water depth and negatively correlated with reservoir age (*P* < 0.05). RDA was employed to determine the correlation between the physicochemical properties of the sediments and the archaeal community composition. The sedimentary physicochemical characteristics, dam engineering, and geographical factors in the first two axes of the RDA analysis can explain 52.9% and 12.8% of the total variance in sediment archaeal OTU composition, respectively (Fig 7). The sedimentary physicochemical characteristics, dam engineering, and geographical factors, including NH_4_^+^-N (F = 5.0; P = 0.024, 499 permutations), reservoir age (F = 3.1; P = 0.046, 499 permutations), and altitude (F = 4.1; P = 0.04, 499 permutations), significantly contributed to the sediment archaeal community-environment relationship.

**Table 1:** Spearman correlation analysis of archaeal communities with environmental factors.

	T	Sand	Silt	pH	Moisture	TC	TOC	TP	TN	NH_4_+-N	NO_3_^-^-N	NO_2_^-^-N	Altitude	Water depth	C/N	HRT	Ra
**Archaeal abundance**	0.479*	-0.493*	0.406*	-0.148	0.169	0.552*	0.331	0.436*	0.459*	-0.051	-0.125	0.036	-0.419*	-0.127	-0.045	-0.270	0.259
**OTU**	0.395*	0.102	-0.204	-0.111	-0.063	-0.070	-0.065	-0.010	0.120	0.166	-0.160	-0.133	-0.286	-0.325	-0.416*	-0.320	0.174
**Shannon**	0.390*	0.179	-0.279	-0.242	-0.074	-0.033	-0.011	-0.137	0.088	0.258	0.009	0.017	-0.485*	-0.335	-0.164	-0.173	0.323
**Simpson**	0.382*	0.276	-0.429*	-0.250	-0.190	-0.207	-0.035	-0.284	-0.083	0.255	-0.051	-0.054	-0.427*	-0.202	0.060	-0.086	0.117
**Chao 1**	0.274	-0.064	-0.095	-0.225	0.034	0.058	0.019	0.115	0.258	0.209	-0.110	-0.021	-0.312	-0.259	-0.409*	-0.259	0.206
***Thaumarchaeota***	0.207	-0.048	-0.074	-0.138	-0.129	-0.002	0.051	0.062	0.138	0.230	-0.381*	-0.291	-0.163	0.232	-0.003	-0.017	-0.375*
***Euryarchaeota***	-0.209	0.223	-0.068	0.308	-0.075	-0.240	-0.176	-0.168	-0.269	-0.384*	0.123	0.092	0.267	-0.207	0.115	-0.024	0.212
***Crenarchaeota***	0.038	-0.172	0.221	-0.294	0.225	0.335	0.230	0.131	0.329	0.003	0.222	0.456*	-0.233	-0.180	-0.356	0.068	0.525*
***Nitrososphaeria***	0.196	-0.052	-0.075	-0.139	-0.120	0.000	0.044	0.065	0.139	0.220	-0.284	-0.295	-0.152	0.221	-0.012	-0.024	-0.377*
***Methanomicrobia***	-0.294	0.118	0.055	0.377*	0.017	-0.067	-0.119	-0.145	-0.218	-0.247	0.194	0.120	0.384*	-0.176	0.101	-0.085	0.110
***Bathyarchaeia***	0.024	-0.178	0.237	-0.267	0.246	0.342	0.235	0.153	0.332	0.013	0.248	0.470*	-0.220	-0.182	-0.358	0.071	0.537*
***Methanobacteria***	0.273	0.357	-0.409*	-0.369*	-0.063	-0.356	-0.060	-0.078	0.076	-0.063	0.219	0.173	-0.412*	-0.186	-0.201	0.285	0.531*
***Thermoplasmata***	-0.267	0.013	0.122	-0.123	-0.065	-0.045	0.169	-0.103	0.123	-0.234	0.153	0.361	0.030	0.360	-0.038	0.475*	-0.111
***Methanosaeta***	0.059	0.033	0.041	0.232	0.053	0.197	-0.239	-0.231	-0.168	-0.158	-0.209	0.008	-0.008	-0.548*	-0.005	-0.761**	0.176
***Methanosarcina***	-0.449*	0.524*	-0.372*	0.286	-0.209	-0.706**	-0.255	-0.183	-0.402*	-0.324	0.191	-0.086	0.416*	0.133	0.236	0.347	-0.082
***Candidatus Nitrososphaera***	-0.201	0.018	-0.034	0.019	-0.117	-0.119	0.120	0.057	0.024	0.201	-0.046	-0.154	0.141	0.491*	0.151	0.344	-0.499*
***Candidatus Methanoperedens***	-0.021	-0.033	-0.016	0.320	-0.090	0.085	-0.263	-0.090	-0.065	0.105	-0.048	-0.138	0.219	-0.413*	-0.187	-0.462*	-0.008
***Methanobacterium***	0.246	0.390*	-0.428*	-0.386*	-0.131	-0.412*	-0.040	-0.047	0.083	-0.046	0.220	0.155	-0.402*	-0.134	-0.196	0.345	0.514*

*Correlation is significant at the 0.05 level

**Correlation is significant at the 0.01 level.

T, water temperature; TC, total carbon content; TOC, total organic carbon content; TN, total nitrogen content; TP, total phosphorous content; C/N, the ratio of TOC to TN; HRT, Hydraulic retention time; Ra, Reservoir age.

## 4 Discussion

### 4.1 The distribution pattern and response to cascade dam construction of archaeal community

Previous studies on the archaeal community have mainly focused on the oceans, estuaries, and natural lakes [39, 40]. Some studies have shown that there is obvious spatial and temporal variation in the abundance of the archaeal community in freshwater [40], but there are limited data on large rivers and reservoirs.

Schwarz et al. [41] found that there was a slight change in the abundance of the archaeal community in Lake Kinneret. Borrel et al. [42] and Ye et al. [16] reported that the abundance of the archaeal community in Lake Pavin and Lake Taihu changed with the depth of sediment stratification. In this study, the abundance of sediment archaeal in the summer and winter of the LRCR was 1.93 × 10^5^–1.03 × 10^6^ and 5.11 × 10^4^–7.90 × 10^5^ copies per gram dry sediment, respectively, which were lower than that in Kinneret Lake (meso-eutrophic), Pavin Lake (oligomesotrophic), and Taihu Lake (eutrophication) [41, 43]. The abundance of the archaeal community in the sediments of the M, GGQ, NZD, and JH reservoirs was significantly higher in the summer than in winter, while that in the XW, MW, and DCS reservoirs was the opposite, which shows that the abundance of the archaeal community in the sediments of different reservoirs has seasonal heterogeneity. At the same time, the abundance of the archaeal community in reservoir sediment was also significantly affected by spatial distribution, which is similar to previous research results on lakes [44]. Correlation analysis suggested that the abundance of archaea was positively correlated with bottom water temperature, silt content, organic matter, TP, and TN, but negatively correlated with sediment concentration and altitude. This further indicates that the quantity of archaeal in the sediments of cascade reservoirs was not only related to the nutritional status of reservoirs but also related to the geographical environment and engineering characteristics of dams. Swan et al. [45] studied the sediments of a hypersaline lake in California and found that the influence of environmental factors on archaea was relatively weak. Jiang et al. [46] showed that an increase in salinity would increase the abundance of archaea. However, in this study, the abundance of archaea in the sediments was generally larger than that in the sediments of the hypersaline lake in California [45]. The potential reason may be that the extreme conditions, such as hypersalinity, were not conducive to the growth of microorganisms, which led to a relatively extreme natural environment dominated by some archaea with high tolerance. Chen et al. [47] showed that the abundance of bacteria in the sediments of the nearshore zone of the Lancang River reservoir system would be increased by exogenous organic carbon, but no specific study on the archaeal community in the sediments of the deep-water area of the reservoir was conducted. The cascade reservoirs of the Lancang River are deep and narrow canyon reservoirs with deep water depths. Affected by the time of reservoir construction and the environmental characteristics of the basin, the nutrient content in the sediment was relatively low, while the ancient bacteria showed great adaptability to this environment. Because of the limitation of sampling frequency and the number of sampling points, the impact of river velocity and reservoir operation on the sediment archaeal community needs further study.

Some previous studies have reported the marked spatial heterogeneity of archaeal community richness and diversity in reservoir and lake sediments [48, 49], but the influence of seasonal variation on archaeal community richness and diversity in reservoir and lake sediments remains unclear. There was also significant spatial variation in different reservoirs in the Lancang River, which was inconsistent with the previous studies of other lakes, reservoirs, and rivers [50, 51]. Schwarz et al. [41] found that the abundance and diversity of the archaeal community in Lake Kinneret sediments show relatively high little seasonal change, while Rodrigues et al. [52] found that seasonal change had a significant impact on the richness and diversity of the archaeal community in Cerrado lake sediment. Previous studies on lakes and single reservoirs have shown that the diversity of archaeal communities is affected by sediment depth, sampling time, and geographical factors [23, 48, 53, 54]. The study on the LRCR shows that the input of allochthonous organic carbon will significantly affect the diversity of the archaeal community in the sediments of the reservoir.

Previous studies have illustrated the evident discrepancy in the archaeal community structure between different freshwater lakes [55–57]. Therefore, the archaeal community structure in this study may be reservoir-specific. Phylogenetic analysis of archaeal communities indicated that the phylum *Thaumarchaeota* (4.94–79.89%) predominated in the sediments of the LRCR. The present study illustrated a remarkable spatial fluctuation of the sediment *Thaumarchaeota*, *Euryarchaeota*, and *Bathyarchaeota* proportions in both summer and winter, further indicating the evident spatial change in the sediment archaeal community structure. Zhang et al. [58] found that *Thaumarchaeota* (72.73%) dominated the sediment archaeal community. Berdjeb et al. [51] even suggested the predominance of the phylum *Thaumarchaeota* in two deep freshwater lakes. Several previous studies have shown the dominance of *Thaumarchaeota* in the sediment archaeal community [56, 57, 59]. This is consistent with the results of the current study. Previous studies have shown that *Thaumarchaeota* is closely related to ammonia oxidation in the environment [60, 61]. The phylum *Thaumarchaeota* contains almost all ammonia-oxidizing archaea and is widely distributed in marine sediments. However, few studies have reported that *Thaumarchaeota* is abundant in the archaeal communities of freshwater sediments, such as lakes. Only Rodrigues et al. [52] showed that *Thaumarchaeota*, as the dominant phylum in the alternation period of the summer and winter, exists in the archaeal community of sediments.

In this study, the distribution patterns and gradients of microorganisms in the sediments of cascade reservoirs along the river were studied on a large spatial scale. The results of RDA sequencing showed that geographic factors (altitude, latitude, and longitude) were the main factors affecting the distribution pattern of microorganisms in the sediments along the river. This finding is consistent with previous findings that the diversity of microbial distribution is due to the unique geographical location and different climatic conditions of the basin [62]. Our study area was located in the Longitudinal Range-Gorge Region (LRGR) in Southwest China [63]. Its natural environment is characterized by a north-south parallel trend of mountains and deep valleys, and the Lancang-Mekong River flows along the canyon from northwest to southeast [64]. Because of its unique geographical conditions and heterogeneous biodiversity, the distribution of microorganisms in the sediments of the area presents diverse characteristics. Some studies have shown that the sediment archaeal communities in Kinneret Lake [41] and Taihu Lake [65] are less affected by seasonal changes, while the sediment of the archaeal community in Cerrado Lake has obvious seasonal changes [52]. In contrast to natural lakes, reservoirs and other artificial water storage facilities are affected by the joint operation of cascade hydropower stations, the time of reservoir construction, and the method of water release, which contribute to the complex characteristics of river sediment. XW and NZD are high dams and large reservoirs with annual regulation capacity in the cascade reach of the Lancang River. The reservoirs have stable hydrothermal stratification and high sediment interception capacity. The conversion of water and heat in summer and winter may lead to sediment resuspension.

Compared with a single reservoir, the construction of the cascade reservoir has a more complex influence on the microbial abundance of river sediment. At present, it is impossible to evaluate and analyze the microbial status of natural rivers before cascade development. Although the method of replacing time with space has great advantages in the study of hydrological characteristics of rivers before and after engineering development [66], there were obvious disadvantages in the study of microscopic biological characteristics according to its principle. From the viewpoint of ecology, biological evolution is the result of natural selection. The characteristics of temporal and spatial changes in a short period may not fully explain the variation in the archaeal community in the sediments. There is a significant variation between the different reaches of the same river and different reservoirs.

### 4.2 Metabolic function and influencing factors of archaea in sediments of LRCR

Previous studies have shown that genes related to the degradation of xenobiotic pollutants were highly abundant in the lower reaches of rivers or river sections with intensive human activities, which is consistent with the findings of this study. Other studies have shown that there may be a correlation between the abundance of xenobiotic degradation genes and the rate of xenobiotic biodegradation [67, 68]. Biodegradation-related genes can be used as indicators of the existence of xenobiotic organisms and their metabolites. A high abundance of genes related to the degradation of naphthalene, benzoate, chloroalkanes, and chloroalkenes, and toluene degradation was observed in the cascade reservoirs of the Lancang River, which was accompanied by an increase in the abundance of potential *methanogens*. It has been found that some genera of *methanogens* can also metabolize other organic pollutants in the environment, such as polycyclic aromatic hydrocarbons [69] and toluene [68]. *Methanogenic* archaea can also participate in the degradation of benzene, toluene, and xylene to produce CH_4_ and CO_2_ [70, 71]. However, unlike many previous studies on urban rivers [72, 73], the abundance of PAH degradation genes in the sediments of the Lancang River was not expected, which may be related to the lower human activity in the Lancang River Basin. The biodegradability of PAHs in the sediments of the Lancang River requires further evaluation and research. In addition, genes related to carbon, nitrogen, phosphorus, and metabolism were detected in the sediment samples, indicating that the archaeal community may play an important role in biogenic material metabolism. Pan et al. [74] found that in addition to its previously known metabolic functions, *Bathyarchaeota* may not only have phototrophy and autotrophy but also play an important role in the global nitrogen and sulfur cycles. Farag et al. [40] found that some marine benthic archaea (Lokiarchaeota) have the potential to degrade aliphatic and aromatic hydrocarbons anaerobically; associate with organisms using nitrate, nitrite, and sulfite as electron acceptors; and possibly degrade benzoate genetically, which indicates that there may be a syntrophic relationship between Lokiarchaeota and nitrite-and sulfite-reducing bacteria. This study extends our understanding of the metabolic function of archaea in freshwater sediments. At present, the distribution patterns of archaeal communities in river sediments at different spatial scales, the co-distribution and co-evolutionary mechanism of archaeal communities in sediments and water columns, and the metabolic process of archaeal communities in sediments and their impact on river ecological functions need to be further studied.

Previous studies on freshwater lakes have shown that TP is the main factor affecting the abundance of archaeal communities in sediments [44]. Other studies have shown that the content of organic matter and nutrients in lake sediments will also have an impact on the abundance of archaeal communities [75], which is consistent with the results that carbon and nitrogen sources can significantly affect the number of bacterial communities in the reservoir sediments in this study. Correlation analysis showed that the abundance of the archaeal community in the sediments of the cascade reservoir was significantly correlated with OM, TN, and NH_4_^+^-N (*P* < 0.05), and negatively correlated with the water depth of the reservoir (*P* < 0.05). This shows that carbon and nitrogen, as the main energy sources of archaeal community metabolism in the sediment, can significantly affect the number of archaeal communities in the reservoir sediment, and the negative correlation between the water depth and the abundance of the archaeal community was probably because the cascade reservoirs of the Lancang River were generally canyon-type deep and narrow, and the deeper water depth led to a lower content of dissolved oxygen in the reservoir sediment, which is not conducive to the growth of the archaeal community.

## 5 Conclusion

Our results show that the abundance, diversity, and composition of archaeal communities in the sediments of the cascade reservoirs in the Lancang River have significant spatial and temporal heterogeneity, and the main physiochemical factors of the sediments, the engineering characteristics of the cascade reservoirs, and the geographical environment factors all contribute to the diversity of the archaeal community. *Thaumarchaeota* (47.51%) and *Euryarchaeota* (30.03%) were the dominant phyla in the sediment archaea, and *Crenarchaeota* (20.64%) were also an important part of the archaeal community. The construction of cascade reservoirs leads to diversity in the composition of archaeal communities in sediments. It is very important to understand the biogeochemical processes of microorganisms and strengthen the ecological management of reservoirs to study the microbial ecology of the regulated river ecosystem using sediments. This provides typical data for comparison with other cascade development rivers, which is helpful in increasing the understanding of the microbial ecology of large rivers.

## Supporting information

S1 Fig Share OTUs among sediments for pooled sequences. (TIF)

S2 Fig Abundance distribution map of the first 10 taxa (phylum) with the most significant difference among samples (summer and winter groups). (TIF)

S3 Fig Abundance distribution map of the first 20 taxa (phylum) with the most significant difference among samples (cascade reservoir groups). (TIF)

S4 Fig Abundance distribution map of the first 10 taxa (genus level) with the most significant difference among samples (summer and winter groups). (TIF)

S5 Fig Abundance distribution map of the first 20 taxa (genus level) with the most significant difference among samples (cascade reservoir groups). (TIF)

S1 Table Main characteristics of cascade hydropower stations in Yunnan section of Lancang River. (DOCX)

S2 Table Longitude, latitude, and elevation of sampling sites in Yunnan section of Lancang River. (DOCX)

S3 Table Mean value of environmental variables measured in the sediments of cascade reservoirs. (DOCX)

S4 Table Statistical table of Metastats pairwise comparison test results between samples (summer and winter groups). (DOCX)

S5 Table Statistical table of Metastats pairwise comparison test results between samples (cascade reservoir groups). (DOCX)

S6 Table The relative abundance of PICRUSt-predicted reads annotated to genes for xenobiotic biodegradation and metabolism from each sequencing library (values were normalized by per mileage). (DOCX)
