# Supplementary figures and images for: Spatial-temporal dynamics and influencing factors of archaeal communities in the sediments of Lancang River cascade reservoirs (LRCR), China
Source: PLoS One. 2021 Jun 15;16(6):e0253233. doi: 10.1371/journal.pone.0253233 (PMC8205147; doi:10.1371/journal.pone.0253233)

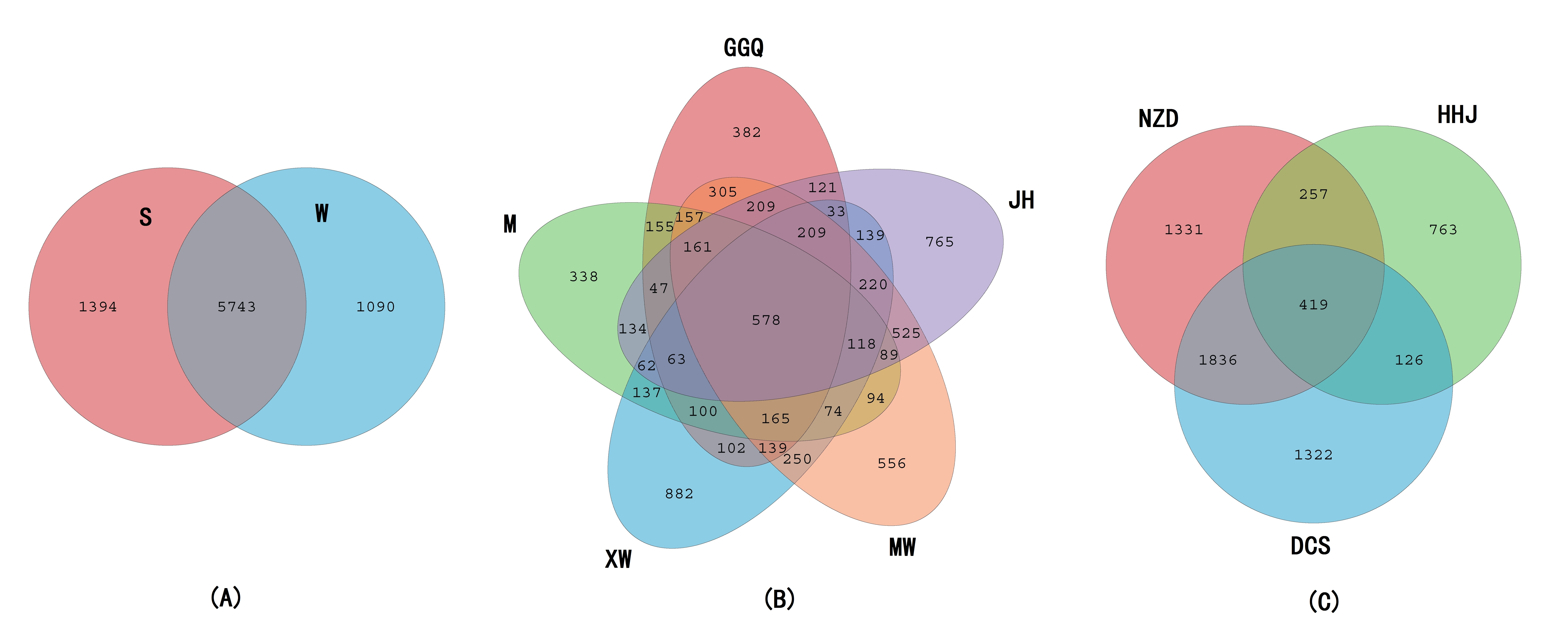

Supplement: S1 Fig — (TIF) [file pone.0253233.s001.tif]

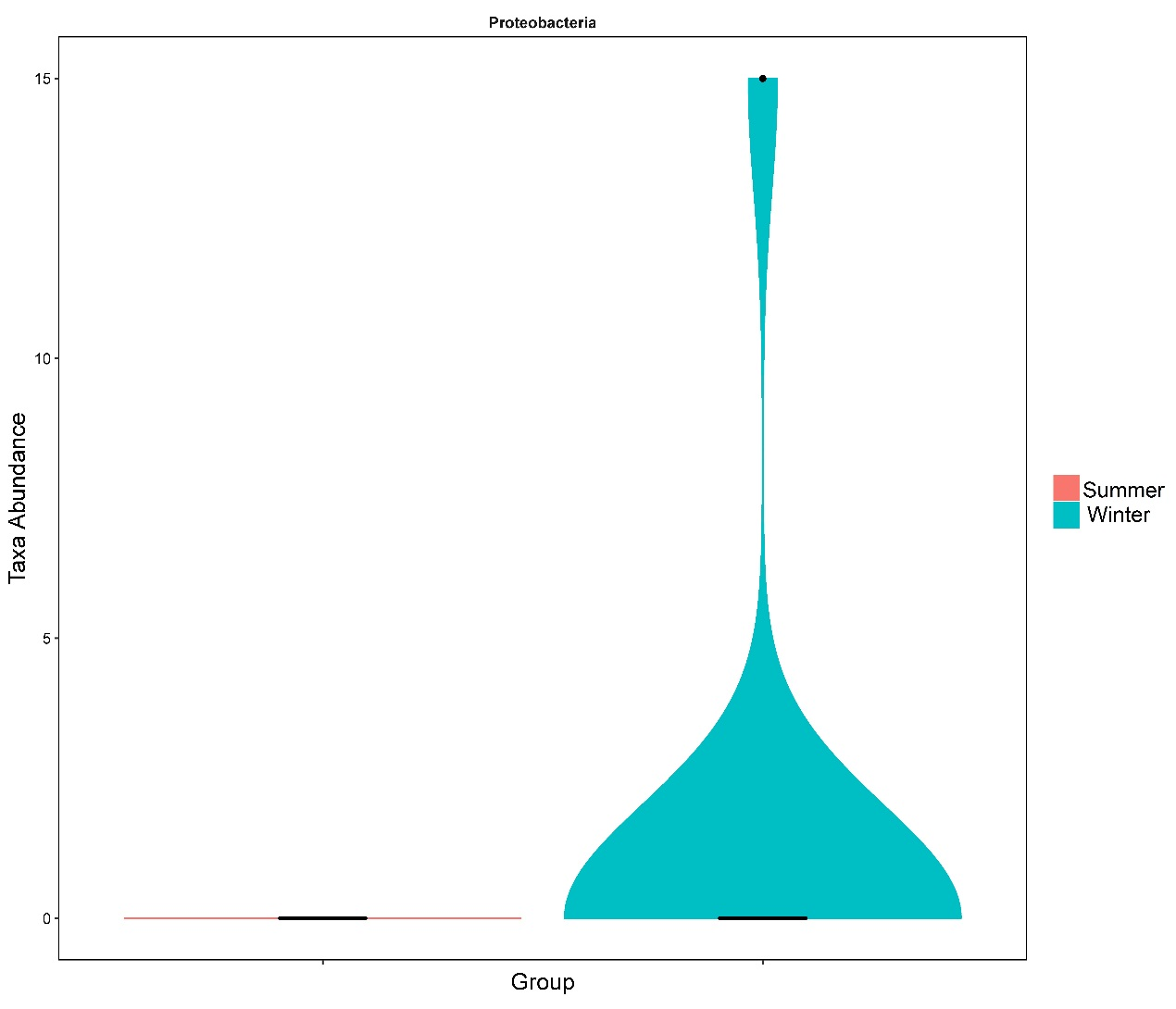

Supplement: S2 Fig — (TIF) [file pone.0253233.s002.tif]

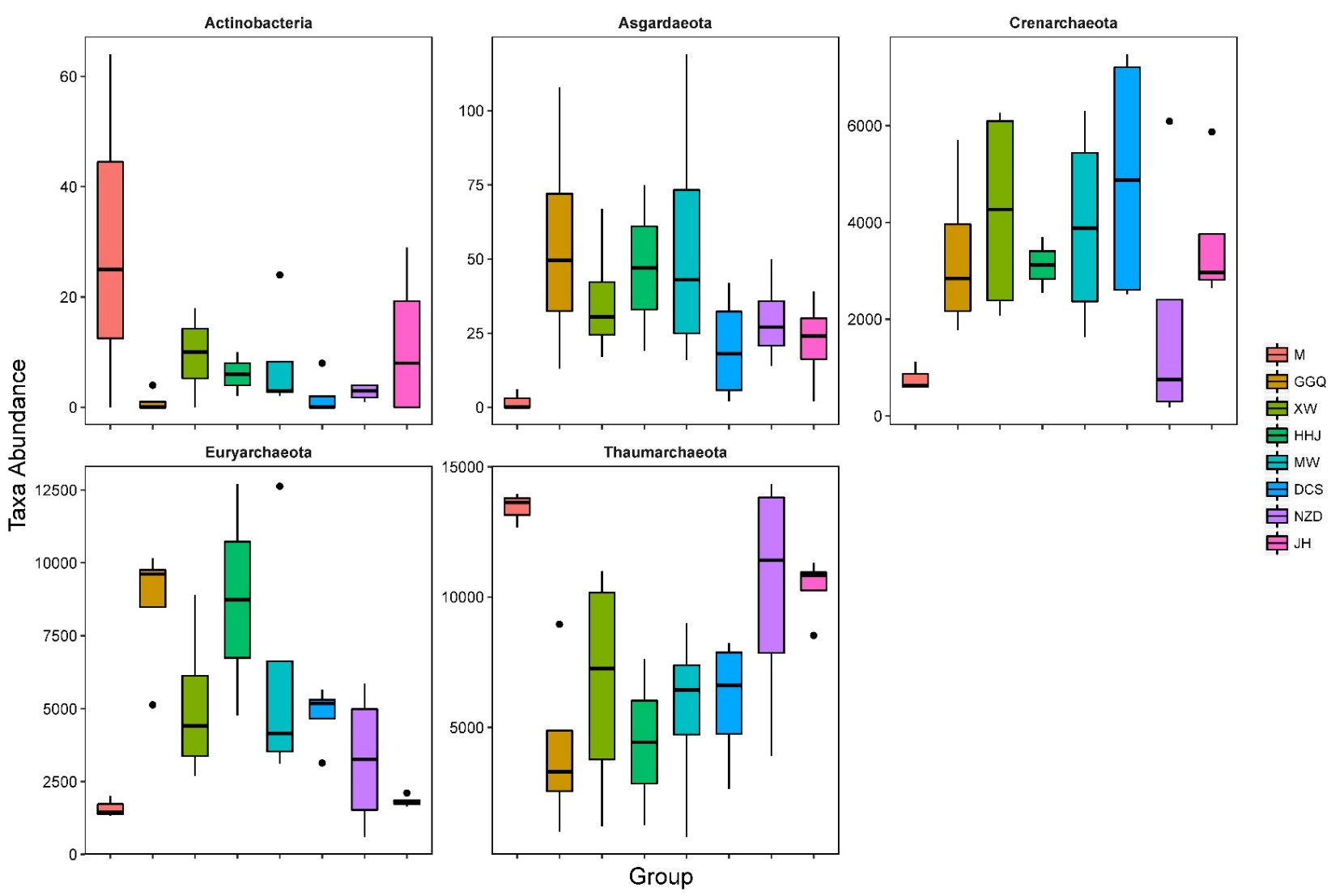

Supplement: S3 Fig — (TIF) [file pone.0253233.s003.tif]

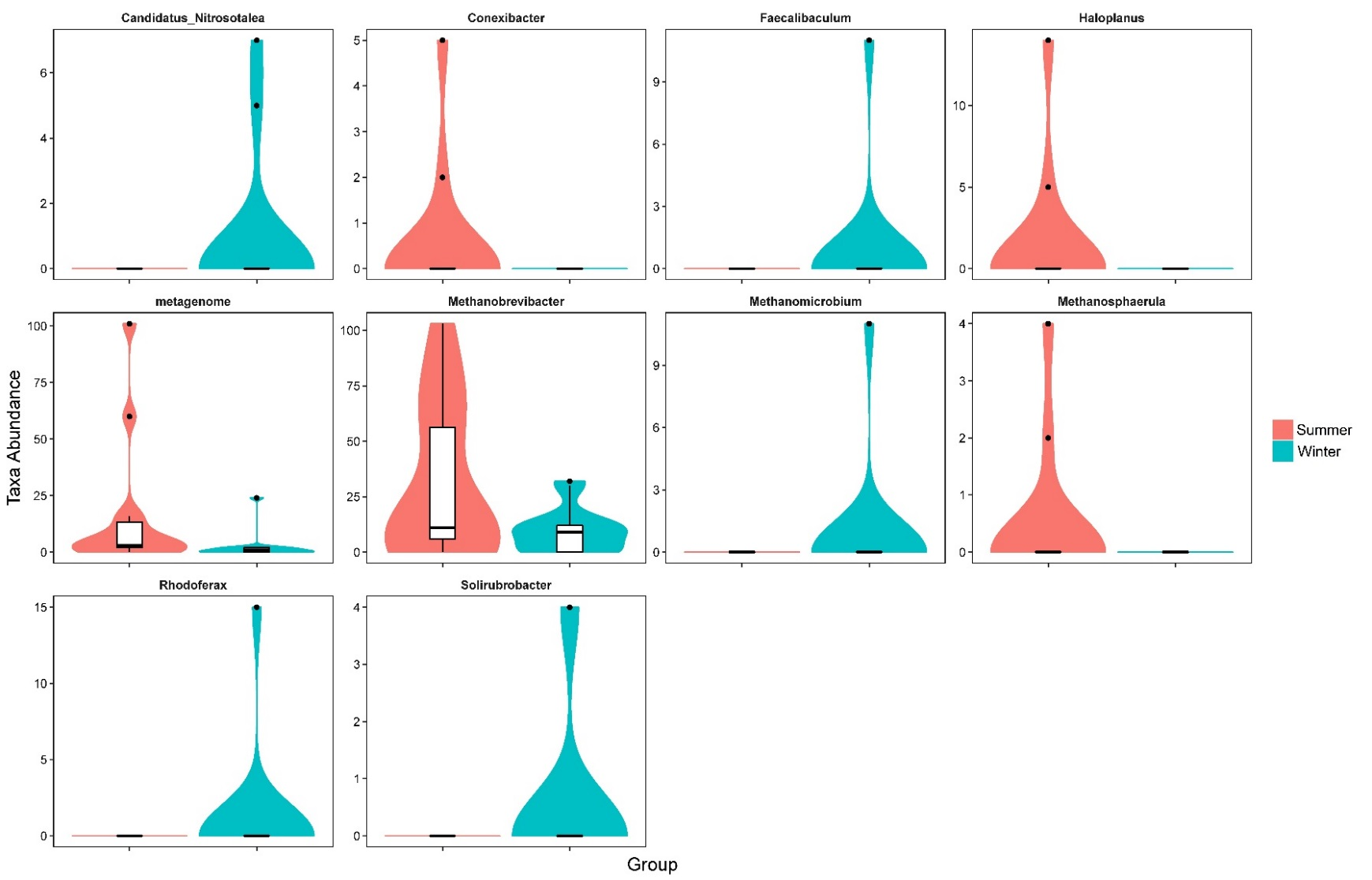

Supplement: S4 Fig — (TIF) [file pone.0253233.s004.tif]

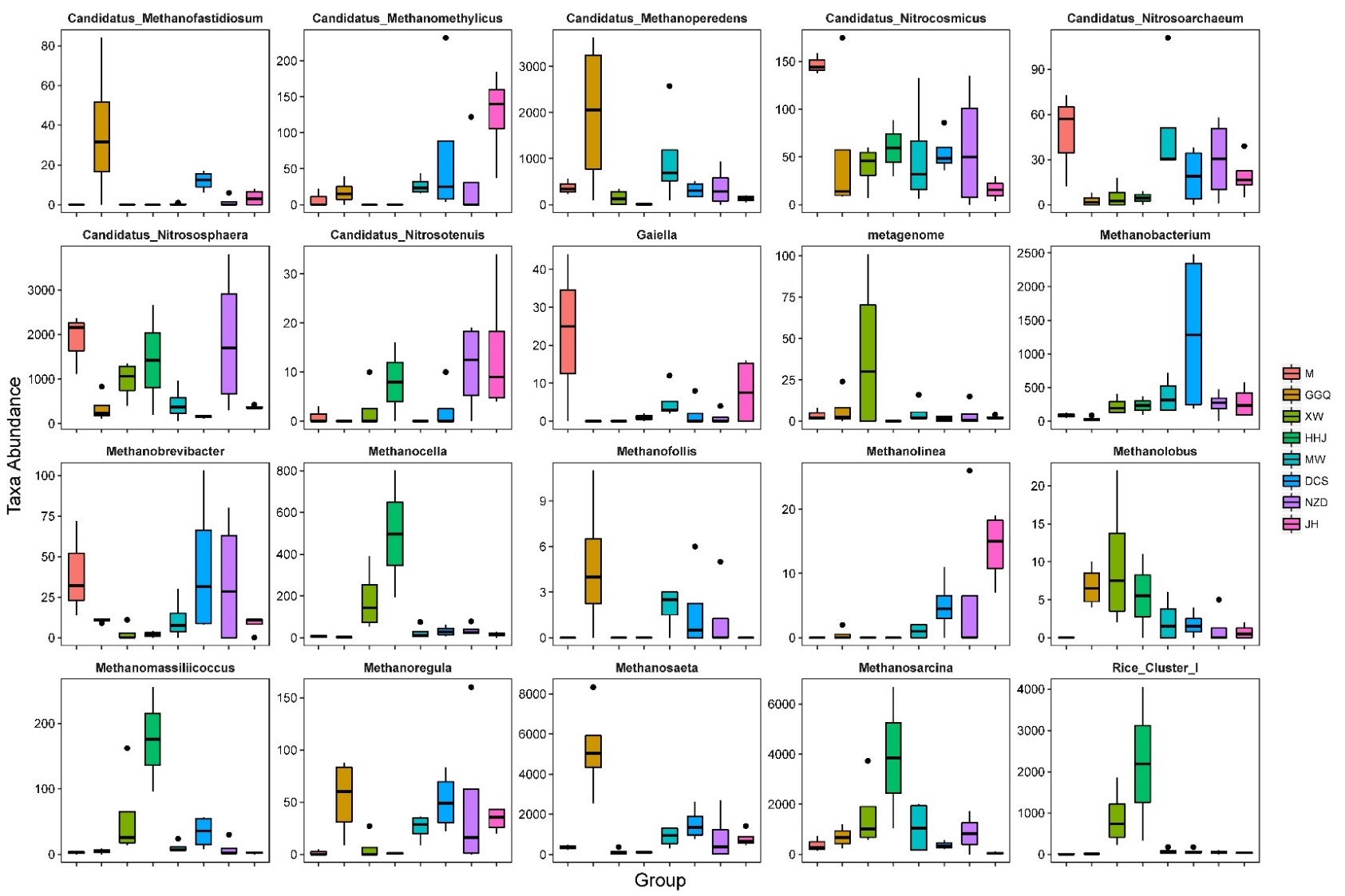

Supplement: S5 Fig — (TIF) [file pone.0253233.s005.tif]
