# Supplementary material for: Spatial-temporal dynamics and influencing factors of archaeal communities in the sediments of Lancang River cascade reservoirs (LRCR), China
Source: PLoS One. 2021 Jun 15;16(6):e0253233. doi: 10.1371/journal.pone.0253233 (PMC8205147; doi:10.1371/journal.pone.0253233)
Supplement: S1 Table — (DOCX) [file pone.0253233.s006.docx]

**S1 Table.** **Main characteristics of Cascade Hydropower Stations** **in Yunnan Section of Lancang River.**

| **Main indicators** | **Miaowei**  **(M)** | **Gongguoqiao (GGQ)** | **Xiaowan**  **(XW)** | **Man wan**  **(MW)** | **Dachaoshan**  **(DCS)** | **Nuozhadu**  **(NZD)** | **Jinghong**  **(JH)** |
| --- | --- | --- | --- | --- | --- | --- | --- |
| **Catchment Area / km^2^** | 93900 | 97200 | 113300 | 114500 | 121000 | 144700 | 149100 |
| **Average Water Flow / (m^3^/s)** | 960 | 985 | 1220 | 1230 | 1340 | 1750 | 1840 |
| **Dam Height / m** | 131 | 130 | 300 | 126 | 110 | 254 | 118 |
| **Reservoir Surface Area / km^2^** | 14.26 | 16.72 | 189.1 | 23.6 | 26.25 | 320 | 32.81 |
| **Backwater Length / km** | 60 | 52 | 178 | 70 | 80 | 210 | 105 |
| **Operating Water Level / m** | 1408 | 1319 | 1236 | 994 | 895 | 807 | 602 |
| **Effective Storage Capacity / (×10^8^m^3^)** | 165 | 120 | 9900 | 257 | 367 | 12300 | 249 |
| **Total Reservoir Volume / (×10^8^m^3^)** | 772 | 510 | 14560 | 920 | 933 | 22400 | 1233 |
| **Hydrological Residence Time / y** | 0.02 | 0.01 | 2.36 | 0.78 | 0.3 | 1.87 | 0.4 |
| **Installed Capacity / MW** | 1400 | 750 | 4200 | 1500 | 1350 | 5850 | 1750 |
| **Regulation Pattern** | Adjusted Weekly | Adjusted Daily | Not Yearly Adjusted | Not Seasonally Adjusted | Not Seasonally Adjusted | Not Yearly Adjusted | Not Seasonally Adjusted |
| **Impoundment time** | 2016 | 2011 | 2008 | 1993 | 2001 | 2011 | 2008 |
