# Supplementary material for: Spatial-temporal dynamics and influencing factors of archaeal communities in the sediments of Lancang River cascade reservoirs (LRCR), China
Source: PLoS One. 2021 Jun 15;16(6):e0253233. doi: 10.1371/journal.pone.0253233 (PMC8205147; doi:10.1371/journal.pone.0253233)
Supplement: S2 Table — (DOCX) [file pone.0253233.s007.docx]

**S2 Table.** **Longitude, latitude, and elevation of sampling sites in Yunnan Section of Lancang River.**

| **Abbreviations** | **Longitude (°E)** | **Latitude (°N)** | **Distance to the frontier (km)** | **Elevation (m)** |
| --- | --- | --- | --- | --- |
| **M01** | 99.14 | 26.2 | 794 | 1382 |
| **M02** | 99.16 | 25.85 | 754 | 1311 |
| **GGQ01** | 99.23 | 25.76 | 732 | 1296 |
| **GGQ02** | 99.33 | 25.59 | 713 | 1259 |
| **XW01** | 99.73 | 24.97 | 605 | 1105 |
| **XW02** | 100.13 | 24.74 | 557 | 1046 |
| **HHJ01** | 100.09 | 24.71 | 548 | 1045 |
| **MW01** | 100.1 | 24.67 | 539 | 1007 |
| **MW02** | 100.4 | 24.63 | 489 | 994 |
| **DCS01** | 100.49 | 24.54 | 476 | 898 |
| **DCS02** | 100.37 | 24.04 | 396 | 857 |
| **NZD01** | 100.05 | 23.2 | 273 | 692 |
| **NZD02** | 100.4 | 22.66 | 191 | 622 |
| **JH01** | 100.58 | 22.5 | 161 | 585 |
| **JH02** | 100.72 | 22.12 | 99 | 564 |
