# Supplementary material for: Spatial-temporal dynamics and influencing factors of archaeal communities in the sediments of Lancang River cascade reservoirs (LRCR), China
Source: PLoS One. 2021 Jun 15;16(6):e0253233. doi: 10.1371/journal.pone.0253233 (PMC8205147; doi:10.1371/journal.pone.0253233)
Supplement: S3 Table — (DOCX) [file pone.0253233.s008.docx]

**S3 Table.** **Mean value of environmental variables measured in the sediments of cascade reservoirs.**

|  | **Abbreviations** | **T**  **(℃)** | **Sand**  **(%)** | **Clay**  **(%)** | **Silt**  **(%)** | **pH** | **Water content (%)** | | **OC**  **(%)** | **TOC**  **(g/kg)** | **TP**  **(mg/kg)** | **TN**  **(mg/kg)** | **NH_4_^+^-N (mg/kg)** | **NO_3_^-^_-_N (mg/kg)** | **NO_2_^-^_-_N (mg/kg)** |
| --- | --- | --- | --- | --- | --- | --- | --- | --- | --- | --- | --- | --- | --- | --- | --- |
| **Summer** | **SM01** | 18.20 | 16.13 | 24.18 | 59.69 | 8.14 | 16.87 | 0.54 | | 11.07 | 743.29 | 1009.49 | 18.47 | 2.45 | 0.06 |
|  | **SGGQ01** | 18.47 | 22.18 | 30.52 | 47.30 | 8.34 | 22.83 | 1.05 | | 10.47 | 550.43 | 745.23 | 16.00 | 1.90 | 0.05 |
|  | **SGGQ02** | 18.60 | 11.54 | 20.92 | 67.54 | 8.41 | 22.73 | 0.49 | | 12.32 | 631.87 | 949.17 | 16.12 | 2.75 | 0.09 |
|  | **SXW01** | 13.43 | 31.00 | 20.48 | 48.52 | 7.86 | 23.23 | 0.26 | | 11.57 | 666.08 | 1297.17 | 13.71 | 3.43 | 0.10 |
|  | **SXW02** | 13.30 | 13.37 | 29.63 | 57.00 | 8.15 | 28.27 | 0.60 | | 17.81 | 894.51 | 1656.31 | 23.99 | 4.04 | 0.09 |
|  | **SHHJ01** | 13.97 | 20.83 | 33.57 | 45.60 | 7.23 | 20.00 | 0.48 | | 15.95 | 799.17 | 960.71 | 15.46 | 2.46 | 0.07 |
|  | **SMW01** | 19.47 | 57.64 | 29.52 | 12.84 | 8.22 | 24.47 | 0.18 | | 9.51 | 613.73 | 732.41 | 32.69 | 4.31 | 0.09 |
|  | **SMW02** | 19.53 | 11.51 | 31.24 | 57.25 | 7.74 | 23.50 | 1.01 | | 16.02 | 806.61 | 1888.54 | 32.97 | 4.54 | 0.10 |
|  | **SDCS01** | 20.37 | 30.79 | 25.82 | 43.39 | 7.79 | 18.17 | 0.39 | | 11.26 | 560.79 | 1030.21 | 21.67 | 2.57 | 0.06 |
|  | **SDCS02** | 20.43 | 14.70 | 29.45 | 55.85 | 7.81 | 26.03 | 0.77 | | 15.14 | 961.08 | 1623.05 | 17.55 | 2.52 | 0.09 |
|  | **SNZD01** | 16.47 | 35.37 | 27.78 | 36.85 | 7.39 | 19.67 | 0.39 | | 12.23 | 474.07 | 978.98 | 20.21 | 2.44 | 0.06 |
|  | **SNZD02** | 16.54 | 21.36 | 29.11 | 49.52 | 8.02 | 25.87 | 0.82 | | 17.53 | 632.80 | 1052.35 | 16.04 | 2.81 | 0.08 |
|  | **SJH01** | 27.80 | 20.68 | 36.56 | 42.77 | 6.99 | 23.13 | 0.52 | | 11.82 | 575.20 | 1030.74 | 14.83 | 2.24 | 0.08 |
|  | **SJH02** | 27.58 | 15.04 | 21.33 | 63.63 | 7.53 | 26.10 | 0.92 | | 20.69 | 764.63 | 1763.30 | 20.33 | 2.87 | 0.11 |
| **Winter** | **WM01** | 9.83 | 26.21 | 33.29 | 40.50 | 8.12 | 31.03 | 0.63 | | 10.17 | 632.93 | 865.23 | 17.82 | 2.18 | 0.05 |
|  | **WM02** | 10.07 | 11.64 | 29.19 | 59.17 | 8.03 | 31.00 | 0.89 | | 11.07 | 650.27 | 1063.82 | 15.32 | 2.45 | 0.06 |
|  | **WGGQ01** | 12.17 | 30.91 | 24.77 | 44.32 | 8.33 | 23.61 | 0.44 | | 11.00 | 485.14 | 785.23 | 17.46 | 2.64 | 0.07 |
|  | **WGGQ02** | 13.30 | 13.53 | 18.97 | 67.50 | 8.40 | 26.47 | 0.89 | | 11.99 | 710.95 | 1012.40 | 17.42 | 2.89 | 0.11 |
|  | **WXW01** | 14.20 | 26.65 | 23.61 | 49.74 | 7.84 | 20.14 | 0.47 | | 12.18 | 522.51 | 1209.67 | 14.26 | 3.54 | 0.12 |
|  | **WXW02** | 14.67 | 12.99 | 18.97 | 68.04 | 8.16 | 34.90 | 0.86 | | 18.11 | 854.67 | 1586.97 | 25.54 | 4.64 | 0.11 |
|  | **WHHJ01** | 14.60 | 13.43 | 32.15 | 54.43 | 7.19 | 34.57 | 0.71 | | 16.79 | 731.87 | 917.85 | 15.58 | 2.86 | 0.07 |
|  | **WMW01** | 15.27 | 59.87 | 24.20 | 15.93 | 8.23 | 29.53 | 0.28 | | 9.87 | 590.31 | 647.77 | 15.75 | 4.18 | 0.09 |
|  | **WMW02** | 15.53 | 11.20 | 22.37 | 66.43 | 7.78 | 33.63 | 1.06 | | 16.92 | 773.28 | 1642.14 | 24.85 | 4.77 | 0.11 |
|  | **WDCS01** | 16.50 | 21.62 | 27.97 | 51.67 | 7.71 | 27.67 | 0.69 | | 10.86 | 527.60 | 1053.27 | 12.70 | 2.51 | 0.11 |
|  | **WDCS02** | 16.37 | 12.02 | 27.92 | 60.06 | 7.77 | 27.07 | 1.07 | | 14.84 | 927.76 | 1485.68 | 19.88 | 4.00 | 0.09 |
|  | **WNZD01** | 19.93 | 22.92 | 36.64 | 40.44 | 7.38 | 19.59 | 0.37 | | 11.90 | 438.99 | 794.30 | 20.35 | 3.15 | 0.11 |
|  | **WNZD02** | 21.70 | 17.05 | 23.04 | 59.91 | 8.09 | 21.78 | 0.85 | | 16.28 | 599.47 | 1031.48 | 24.08 | 2.53 | 0.08 |
|  | **WJH01** | 23.03 | 17.86 | 37.61 | 44.53 | 7.11 | 32.00 | 0.78 | | 12.46 | 554.67 | 838.59 | 30.93 | 2.83 | 0.09 |
|  | **WJH02** | 23.90 | 9.46 | 24.07 | 66.47 | 7.65 | 30.13 | 1.07 | | 19.44 | 732.29 | 1663.82 | 18.65 | 2.68 | 0.10 |
