# Supplementary material for: Spatial-temporal dynamics and influencing factors of archaeal communities in the sediments of Lancang River cascade reservoirs (LRCR), China
Source: PLoS One. 2021 Jun 15;16(6):e0253233. doi: 10.1371/journal.pone.0253233 (PMC8205147; doi:10.1371/journal.pone.0253233)
Supplement: S4 Table — (DOCX) [file pone.0253233.s009.docx]

**S4 Table.** **Statistical table of Metastats pairwise comparison test results between samples (summer and winter groups).**

| **Group** | **Phylum** | **Genus** |
| --- | --- | --- |
| **Summer-Winter** | 1 | 10 |
