# Supplementary material for: Spatial-temporal dynamics and influencing factors of archaeal communities in the sediments of Lancang River cascade reservoirs (LRCR), China
Source: PLoS One. 2021 Jun 15;16(6):e0253233. doi: 10.1371/journal.pone.0253233 (PMC8205147; doi:10.1371/journal.pone.0253233)
Supplement: S5 Table — (DOCX) [file pone.0253233.s010.docx]

**S5 Table.** **Statistical table of Metastats pairwise comparison test results between samples (cascade reservoir groups).**

| **Group** | **Phylum** | **Genus** |
| --- | --- | --- |
| **DCS-GGQ** | 1 | 4 |
| **DCS-HHJ** | 0 | 6 |
| **DCS-JH** | 2 | 6 |
| **DCS-M** | 3 | 7 |
| **DCS-MW** | 0 | 1 |
| **DCS-NZD** | 0 | 2 |
| **DCS-XW** | 0 | 8 |
| **GGQ-HHJ** | 0 | 7 |
| **GGQ-JH** | 2 | 7 |
| **GGQ-M** | 4 | 8 |
| **GGQ-MW** | 0 | 5 |
| **GGQ-NZD** | 2 | 5 |
| **GGQ-XW** | 1 | 11 |
| **HHJ-JH** | 0 | 8 |
| **HHJ-M** | 2 | 5 |
| **HHJ-MW** | 0 | 7 |
| **HHJ-NZD** | 0 | 2 |
| **HHJ-XW** | 0 | 0 |
| **JH-M** | 3 | 7 |
| **JH-MW** | 1 | 5 |
| **JH-NZD** | 0 | 2 |
| **JH-XW** | 1 | 10 |
| **M-MW** | 3 | 7 |
| **M-NZD** | 1 | 3 |
| **M-XW** | 4 | 8 |
| **MW-NZD** | 0 | 1 |
| **MW-XW** | 0 | 10 |
| **NZD-XW** | 0 | 3 |
