# Supplementary material for: Spatial-temporal dynamics and influencing factors of archaeal communities in the sediments of Lancang River cascade reservoirs (LRCR), China
Source: PLoS One. 2021 Jun 15;16(6):e0253233. doi: 10.1371/journal.pone.0253233 (PMC8205147; doi:10.1371/journal.pone.0253233)
Supplement: S6 Table — (DOCX) [file pone.0253233.s011.docx]

**S6 Table.** **The relative abundance of PICRUSt-predicted reads annotated to genes for xenobiotic biodegradation and metabolism from each sequencing library (Values were normalized by per mileage).**

| **Category** | **Compounds metabolism** | **M01** | **M02** | **GGQ**  **01** | **GGQ**  **02** | **XW01** | **XW02** | **HHJ01** | **MW01** | **MW02** | **DCS**  **01** | **DCS**  **02** | **NZD**  **01** | **NZD**  **02** | **JH01** | **JH02** |
| --- | --- | --- | --- | --- | --- | --- | --- | --- | --- | --- | --- | --- | --- | --- | --- | --- |
| **Xenobiotics biodegradation** | **Aminobenzoate** | 39.49 | -- | 24.29 | 26.99 | 29.42 | 38.78 | 35.46 | 27.52 | 29.07 | 26.41 | 31.15 | 27.07 | 30.41 | 38.27 | 38.24 |
|  |  | 47.95 | 46.03 | 32.36 | 25.73 | 33.99 | 31.40 | 36.51 | 13.87 | 36.59 | 26.45 | 27.96 | 42.59 | 31.95 | 31.59 | 34.19 |
|  | **Atrazine** | 17.21 | -- | 3.69 | 4.76 | 10.59 | 14.67 | 10.41 | 9.21 | 7.16 | 8.77 | 10.31 | 4.38 | 10.50 | 15.27 | 14.35 |
|  |  | 20.82 | 20.05 | 10.89 | 1.30 | 1.72 | 4.64 | 1.47 | 1.09 | 8.22 | 2.77 | 5.51 | 19.46 | 15.01 | 9.06 | 12.86 |
|  | **Benzoate** | 94.34 | -- | 45.26 | 48.29 | 68.60 | 91.41 | 82.22 | 60.77 | 65.58 | 68.01 | 81.27 | 60.50 | 66.87 | 95.10 | 93.13 |
|  |  | 116.00 | 109.26 | 71.30 | 50.45 | 67.46 | 68.16 | 53.13 | 41.86 | 86.06 | 57.99 | 66.29 | 103.17 | 78.35 | 76.11 | 82.67 |
|  | **Caprolactam** | 19.12 | -- | 11.36 | 10.87 | 15.71 | 20.42 | 20.84 | 13.03 | 17.94 | 16.01 | 19.53 | 17.91 | 13.93 | 21.93 | 22.29 |
|  |  | 24.82 | 23.13 | 15.60 | 15.43 | 19.93 | 19.32 | 9.17 | 7.65 | 25.24 | 18.46 | 19.97 | 20.67 | 16.00 | 21.24 | 19.42 |
|  | **Chloroalkane and chloroalkene** | 74.12 | -- | 27.03 | 32.65 | 48.25 | 64.84 | 51.97 | 46.47 | 37.59 | 48.45 | 57.05 | 31.21 | 51.93 | 68.21 | 64.63 |
|  |  | 88.06 | 84.10 | 52.28 | 21.54 | 31.86 | 32.36 | 39.74 | 34.81 | 45.65 | 24.90 | 32.67 | 81.51 | 61.42 | 44.18 | 57.13 |
|  | **Chlorocyclohexane and chlorobenzene** | 17.36 | -- | 4.07 | 5.10 | 10.72 | 14.82 | 10.56 | 9.48 | 7.51 | 8.95 | 10.47 | 4.83 | 10.80 | 15.66 | 14.68 |
|  |  | 21.01 | 20.55 | 11.03 | 1.81 | 2.10 | 4.82 | 1.81 | 1.39 | 8.73 | 3.62 | 5.77 | 19.89 | 15.30 | 9.47 | 13.02 |
|  | **Dioxin** | 0.35 | -- | 0.16 | 0.14 | 0.53 | 0.95 | 0.83 | 0.69 | 0.37 | 2.12 | 2.47 | 0.77 | 0.70 | 0.85 | 0.63 |
|  |  | 0.25 | 0.40 | 0.14 | 0.18 | 2.03 | 2.58 | 5.20 | 5.43 | 0.48 | 0.42 | 0.37 | 0.41 | 0.07 | 0.23 | 0.22 |
|  | **Ethylbenzene** | 1.39 | -- | 2.13 | 2.98 | 1.28 | 1.47 | 1.99 | 2.39 | 1.41 | 1.07 | 0.82 | 1.33 | 2.52 | 0.46 | 0.56 |
|  |  | 0.80 | 0.93 | 1.69 | 0.78 | 5.84 | 3.18 | 12.19 | 2.00 | 0.88 | 1.27 | 0.59 | 1.03 | 0.32 | 0.34 | 0.32 |
|  | **Fluorobenzoate** | 0.08 | -- | 0.00 | 0.00 | 0.03 | 0.04 | 0.08 | 0.08 | 0.13 | 0.03 | 0.02 | 0.04 | 0.12 | 0.05 | 0.08 |
|  |  | 0.07 | 0.22 | 0.02 | 0.00 | 0.07 | 0.10 | 0.15 | 0.09 | 0.05 | 0.04 | 0.04 | 0.09 | 0.01 | 0.05 | 0.01 |
|  | **Naphthalene** | 36.25 | -- | 11.59 | 14.74 | 22.68 | 31.07 | 23.07 | 21.35 | 16.21 | 19.10 | 22.13 | 11.59 | 24.30 | 31.77 | 30.07 |
|  |  | 43.10 | 42.14 | 24.68 | 7.35 | 9.83 | 12.72 | 15.93 | 5.01 | 18.24 | 8.77 | 12.44 | 40.41 | 30.66 | 19.29 | 26.77 |
|  | **Nitrotoluene** | 224.44 | -- | 115.83 | 130.96 | 157.63 | 208.40 | 176.04 | 142.77 | 143.09 | 147.90 | 177.60 | 126.58 | 160.09 | 213.78 | 208.11 |
|  |  | 272.09 | 254.32 | 174.22 | 115.94 | 127.18 | 129.41 | 108.09 | 101.98 | 178.84 | 111.52 | 134.34 | 243.42 | 184.30 | 159.73 | 189.45 |
|  | **Polycyclic aromatic hydrocarbon** | 5.30 | -- | 12.20 | 14.66 | 5.93 | 5.83 | 9.99 | 8.95 | 9.85 | 5.75 | 6.33 | 10.73 | 9.47 | 3.68 | 4.92 |
|  |  | 4.09 | 3.00 | 8.86 | 11.36 | 23.84 | 11.94 | 32.23 | 9.59 | 10.99 | 10.00 | 8.96 | 2.39 | 0.39 | 6.53 | 4.38 |
|  | **Styrene** | 0.03 | -- | 0.01 | 0.02 | 0.20 | 0.41 | 0.40 | 0.20 | 0.14 | 0.16 | 0.12 | 0.08 | 0.21 | 0.05 | 0.21 |
|  |  | 0.21 | 0.44 | 0.05 | 0.02 | 0.76 | 1.24 | 2.74 | 0.11 | 0.06 | 0.05 | 0.05 | 0.07 | 0.02 | 0.09 | 0.03 |
|  | **Toluene** | 73.51 | -- | 31.03 | 32.51 | 52.90 | 70.50 | 62.49 | 45.03 | 50.95 | 50.60 | 60.90 | 44.69 | 49.76 | 74.44 | 72.57 |
|  |  | 90.95 | 84.61 | 53.40 | 33.00 | 44.80 | 48.45 | 23.33 | 27.96 | 66.80 | 42.12 | 51.10 | 80.63 | 61.68 | 59.78 | 64.63 |
|  | **Xylene** | 0.01 | -- | 0.00 | 0.00 | 0.05 | 0.07 | 0.05 | 0.00 | 0.05 | 0.00 | 0.02 | 0.01 | 0.02 | 0.00 | 0.07 |
|  |  | 0.07 | 0.18 | 0.01 | 0.00 | 0.00 | 0.06 | 0.02 | 0.01 | 0.01 | 0.00 | 0.01 | 0.01 | 0.02 | 0.04 | 0.00 |
| **Metabolism of major elements and their compounds** | **Nitrogen** | 173.79 | -- | 85.92 | 98.18 | 121.95 | 161.14 | 139.02 | 116.43 | 111.02 | 126.69 | 150.80 | 100.75 | 128.98 | 167.93 | 161.24 |
|  |  | 206.79 | 195.34 | 131.96 | 81.51 | 116.76 | 108.21 | 122.84 | 119.70 | 138.72 | 89.88 | 103.48 | 188.54 | 140.49 | 123.00 | 142.89 |
|  | **Carbohydrate** | 35.76 | -- | 11.36 | 14.03 | 21.87 | 29.85 | 21.45 | 20.51 | 15.09 | 23.53 | 28.25 | 12.36 | 23.29 | 33.08 | 30.63 |
|  |  | 42.91 | 41.11 | 24.55 | 10.22 | 6.62 | 9.72 | 6.05 | 19.47 | 18.78 | 8.88 | 13.02 | 39.97 | 30.39 | 19.52 | 27.38 |
|  | **Phosphonate and phosphinate** | 18.04 | -- | 5.27 | 6.95 | 12.15 | 17.33 | 13.02 | 11.22 | 8.50 | 9.94 | 11.09 | 5.48 | 12.56 | 15.80 | 14.86 |
|  |  | 21.31 | 20.41 | 11.98 | 1.61 | 8.09 | 12.13 | 19.95 | 2.39 | 8.94 | 3.41 | 5.96 | 20.22 | 15.07 | 9.21 | 13.17 |
|  | **Sulfur** | 42.50 | -- | 28.75 | 31.81 | 33.18 | 42.15 | 41.15 | 31.73 | 36.54 | 35.16 | 41.67 | 35.06 | 34.71 | 43.09 | 42.58 |
|  |  | 49.91 | 46.17 | 35.57 | 28.73 | 46.18 | 37.74 | 42.31 | 23.87 | 45.39 | 33.03 | 35.44 | 44.02 | 31.67 | 37.20 | 37.19 |
|  | **Inorganic ion transport and metabolism** | 93.56 | -- | 44.74 | 50.85 | 64.53 | 86.75 | 72.34 | 59.10 | 57.17 | 63.06 | 75.47 | 50.51 | 66.02 | 89.54 | 86.25 |
|  |  | 113.22 | 106.18 | 70.92 | 45.89 | 52.10 | 55.31 | 50.76 | 52.41 | 72.11 | 43.93 | 53.82 | 101.72 | 76.57 | 65.08 | 78.00 |
